# Genital Chlamydia Prevalence in Europe and Non-European High Income Countries: Systematic Review and Meta-Analysis

**DOI:** 10.1371/journal.pone.0115753

**Published:** 2015-01-23

**Authors:** Shelagh M. Redmond, Karin Alexander-Kisslig, Sarah C. Woodhall, Ingrid V. F. van den Broek, Jan van Bergen, Helen Ward, Anneli Uusküla, Björn Herrmann, Berit Andersen, Hannelore M. Götz, Otilia Sfetcu, Nicola Low

**Affiliations:** 1 Institute of Social and Preventive Medicine, University of Bern, Finkenhubelweg 11, CH‐3012, Bern, Switzerland; 2 HIV & STI Department, National Centre for Infectious Disease Surveillance and Control, Public Health England, 61 Colindale Avenue, London, NW9 5EQ, United Kingdom; 3 Unit of Epidemiology and Surveillance, RIVM/Centre for Infectious Disease Control Netherlands, PO Box 1, 3720 BA, Bilthoven, Netherlands; 4 University of Amsterdam, 1012 WX Amsterdam, Netherlands and STI AIDS Netherlands, Keizersgracht 390, 1016 GB, Amsterdam, Netherlands; 5 Infectious Diseases Epidemiology, School of Public Health, Imperial College London, St Mary’s Campus, Norfolk Place, London, W2 1PG, United Kingdom; 6 Department of Public Health, University of Tartu, Ravila 19, 50411, Tartu, Estonia; 7 Department of Clinical Microbiology, Uppsala University Hospital, SE‐751 85, Uppsala, Sweden; 8 Department of Public Health Interventions, Randers Hospital, Skovlyvej, 8900, Randers, Denmark; 9 Department of Infectious Disease Control, Municipal Public Health Service Rotterdam-Rijnmond, PO Box 70032, 3000 LP, Rotterdam, Netherlands; 10 European Centre for Disease Prevention and Control (ECDC), Tomtebodavägen 11 A, 17183, Stockholm, Sweden; University of California Merced, UNITED STATES

## Abstract

**Background:**

Accurate information about the prevalence of Chlamydia trachomatis is needed to assess national prevention and control measures.

**Methods:**

We systematically reviewed population-based cross-sectional studies that estimated chlamydia prevalence in European Union/European Economic Area (EU/EEA) Member States and non-European high income countries from January 1990 to August 2012. We examined results in forest plots, explored heterogeneity using the I^2^ statistic, and conducted random effects meta-analysis if appropriate. Meta-regression was used to examine the relationship between study characteristics and chlamydia prevalence estimates.

**Results:**

We included 25 population-based studies from 11 EU/EEA countries and 14 studies from five other high income countries. Four EU/EEA Member States reported on nationally representative surveys of sexually experienced adults aged 18–26 years (response rates 52–71%). In women, chlamydia point prevalence estimates ranged from 3.0–5.3%; the pooled average of these estimates was 3.6% (95% CI 2.4, 4.8, I^2^ 0%). In men, estimates ranged from 2.4–7.3% (pooled average 3.5%; 95% CI 1.9, 5.2, I^2^ 27%). Estimates in EU/EEA Member States were statistically consistent with those in other high income countries (I^2^ 0% for women, 6% for men). There was statistical evidence of an association between survey response rate and estimated chlamydia prevalence; estimates were higher in surveys with lower response rates, (p = 0.003 in women, 0.018 in men).

**Conclusions:**

Population-based surveys that estimate chlamydia prevalence are at risk of participation bias owing to low response rates. Estimates obtained in nationally representative samples of the general population of EU/EEA Member States are similar to estimates from other high income countries.

## Introduction

Surveys of the population prevalence of *Chlamydia trachomatis* infections (commonly known as chlamydia) can provide information about the need for measures to prevent and control infection. *C. trachomatis* is the most commonly reported sexually transmitted infection (STI) and the most commonly reported of all notifiable infections in Europe and the USA [1, 2]. *C. trachomatis* causes infection in the lower genital tract in women and men, which can result in upper genital tract complications and transmission of infection during pregnancy and labour [3, 4]. *C. trachomatis* also increases susceptibility to, and infectiousness of, HIV infection [5]. Chlamydia prevalence data for adults aged around 25 years and younger are particularly useful for planning control measures because young adults are affected most [3]. Health authorities in some European and other high income countries recommend screening in this age group to allow both early treatment of asymptomatic infection and the prevention of long term complications [6–9].

National surveillance data report on diagnosed cases of chlamydia infection and reported rates vary widely; from two to 600 per 100,000 population in Europe [1]. These figures cannot be used as estimates of population prevalence, however. Chlamydia infections are mostly asymptomatic and rates of reported infection largely differences in levels of chlamydia testing between countries. Cross-sectional surveys of a representative sample of the general population (population-based surveys) [10] provide less biased estimates of the prevalence of a condition at a particular time than surveys of attenders at health care settings. Participation bias can, however, distort estimates of prevalence in any survey whenever there is incomplete participation [11]. Participation bias is more severe when the prevalence of the condition is low [12] and when participation rates are low, which is likely in surveys of sensitive subjects such as sexual behaviour and STI [12]. In several studies of chlamydia infection participants had higher levels of demographic characteristics or behaviours associated with chlamydia than non-participants [13–15], which would over-estimate prevalence.

National estimates of chlamydia prevalence in cross-sectional population-based surveys vary considerably, even between countries with similar levels of social and economic development [14, 16–19]. Differences in chlamydia prevalence between countries could represent real differences in sexual behaviour patterns and chlamydia control efforts, but might also result from variations in study design and participation rates. The primary objective of this study was to systematically review studies reporting chlamydia prevalence in adult women and men in the general population of the European Union and European Economic Area (EU/EEA). A secondary objective was to investigate the association between survey response rate and estimated chlamydia prevalence in both EU/EEA and other high income countries [20].

## Methods

We conducted a systematic review using a predefined protocol (S1 Text) and reported it in accordance with the guidelines on Preferred Items for the Reporting of Systematic Reviews and Meta-Analyses (PRISMA) [21]. The study is part of a project funded by the European Centre for Disease Control and Prevention, for which a technical report describes the results of a group of literature reviews about chlamydia epidemiology and control [22].

### Inclusion and exclusion criteria

Eligible studies designs were: cross-sectional surveys that used population-based sampling methods and tested genital specimens from adult women and men for *C. trachomatis*. Studies with the following characteristics were excluded: serological studies and studies sampling only from extra-genital sites; participant age below 13 years; data published in letters, commentaries and editorials. We considered the following specific groups as part of the general population: school students if the sampling frame included all schools in the country or in a sub-national geographic region of a country; and military recruits in countries with compulsory military conscription.

The review focussed on adults in EU/EEA Member States at the time of the first database search. We included the following countries to improve the generalisability of our findings and statistical power of our analyses: non-EU/EEA countries in Europe; high income countries, as defined by the Organisation for Economic Cooperation and Development (OECD) [20].

### Data sources and searches

We searched Ovid Medline, Embase, Popline and The Cochrane Library from January 1990 to 17^th^ October 2011 without language restrictions and updated the search on 17^th^ August 2012. Search strategies, adapted for each search engine, included terms for “chlamydia infection” and “prevalence” and individual names of EU/EEA Member States, or “Europe”, or the non-European high income countries Australia, Canada, Israel, Japan, Korea, New Zealand and USA [20]. In addition we searched reference lists of potentially eligible studies and asked experts if they were aware of other studies. For countries with no publications identified in the first search we then used only the country name and the free text term “chlamydia” to find further publications. We included additional data from primary studies included in the review even if the additional publications were published after the search deadline. S1 Text includes the full search strategy.

### Study selection

Two suitably qualified reviewers (SR, KA-K) screened the titles and abstracts of all identified articles independently. The full text of potentially eligible studies was retrieved and two reviewers (SR, KA-K) independently assessed each against predefined inclusion criteria. Studies were translated where necessary. A third reviewer (NL) resolved differences between reviewers if necessary.

### Data extraction and quality assessment

Two reviewers (SR, KA-K, or SW) extracted data independently in duplicate onto standardised piloted forms in EpiData (EpiData Association, Odense, Denmark). If multiple publications were associated with a study, we extracted data from the primary publication first (assigned as the publication with the most detailed description of the survey methods). Data reported in the primary publication were used in the case of inconsistencies. The two reviewers compared the extracted data and resolved differences by discussion. If there was still a discrepancy, a third reviewer (NL) adjudicated. We did not contact authors for additional information.

The following information was extracted: study design; country; study population (sexually experienced only or all participants) and setting (national or sub-national); demographic characteristics; numbers eligible, invited and participating; numbers excluded with reasons; number with *C. trachomatis* detected; diagnostic test method; estimated prevalence and 95% confidence intervals (CI) reported in the study.

We used published guidelines for cross-sectional prevalence surveys to assess the risk of bias related to methodological aspects of included studies [11]. Two reviewers (SR, KA-K, or SW) assessed each study independently. Discrepancies were resolved by discussion or adjudication (NL). The items assessed included: representativeness of the target and source populations; similarity of responders and non-responders; achievement of planned sample size; use of standardised data collection methods; appropriateness of statistical methods; and response rate [11]. We pre-specified criteria to determine whether each feature had been adequately addressed, not adequately addressed, or if there was insufficient information to decide. The guideline defined an adequate response rate as >80% [11]. Few studies attained this level so we also recorded those with response rates of >60% and >70%.

### Data synthesis and analysis

We analysed data for women and men separately. First, we estimated chlamydia prevalence using the number of positive chlamydia tests and the number of people tested. Where authors of included studies reported stratified sampling methods we used the published point estimate and 95% CI. Where simple random sampling was done and data were available, we calculated chlamydia prevalence (with binomial 95% CI).

We used forest plots to examine estimates of chlamydia prevalence. The I^2^ statistic expressed the percentage of variation between estimates in different studies resulting from factors other than random variation [23]. As a guide, I^2^ values above 25%, 50% and 75% are suggested as evidence of mild, moderate and severe between study heterogeneity. Low values of the I^2^ statistic suggest that variability between estimates is compatible with random variation [23]. Where there was evidence of moderate or severe heterogeneity, we explored reasons for this by stratifying studies in pre-defined groups: age ≤25 years; geographic coverage (national or sub-national); and study population analysed (all adults or sexually experienced adults only). Where appropriate, we pooled estimates using random effects meta-analysis to estimate the average of the study estimates and their 95% CI.

We calculated a response rate for each study, using an algorithm to define numerators and denominators consistent with recommendations of the Council of American Survey Research Organisations (CASRO) [24, 25]. Where available, the numerator was the number of people providing a sample for chlamydia testing and the denominator was the number of eligible subjects asked to participate, provide a sample, or sent an invitation for testing. If the study report did not include these numbers we used the number of samples tested, followed by the number of test results used in the analysis as the numerator and the number of eligible people as the denominator. We used the published response rate in studies that used complex sampling methods and post-stratification weighting. It was not possible to calculate a response rate in studies in which the group asked to participate is then asked if they have ever had sexual intercourse and chlamydia testing is restricted to those who are sexually experienced. In such studies, the calculated response rate is underestimated.

We used meta-regression to examine the linear association between estimated chlamydia prevalence in ≤25 year old women and men and the calculated response rate. We applied the sex-specific response rate for the whole study to this age group because most study reports did not report age-specific response rates. In these analyses, the I^2^ statistic represents the percentage of heterogeneity due to factors other than sampling error after taking into account the association between prevalence and response rate. We also used meta-regression to analyse the association between estimated chlamydia prevalence and the following binary variables: sex (women versus men), age (≤25 years versus >25 years), geographical setting (national versus sub-national) and response rate as reported in the included studies (<60% versus ≥60%). We included a term for the individual study in the model when observations from the same study were not independent. All analyses were done using Stata statistical software (Stata 11, StataCorp, Austin, Texas, USA).

## Results

**Figure 1:**
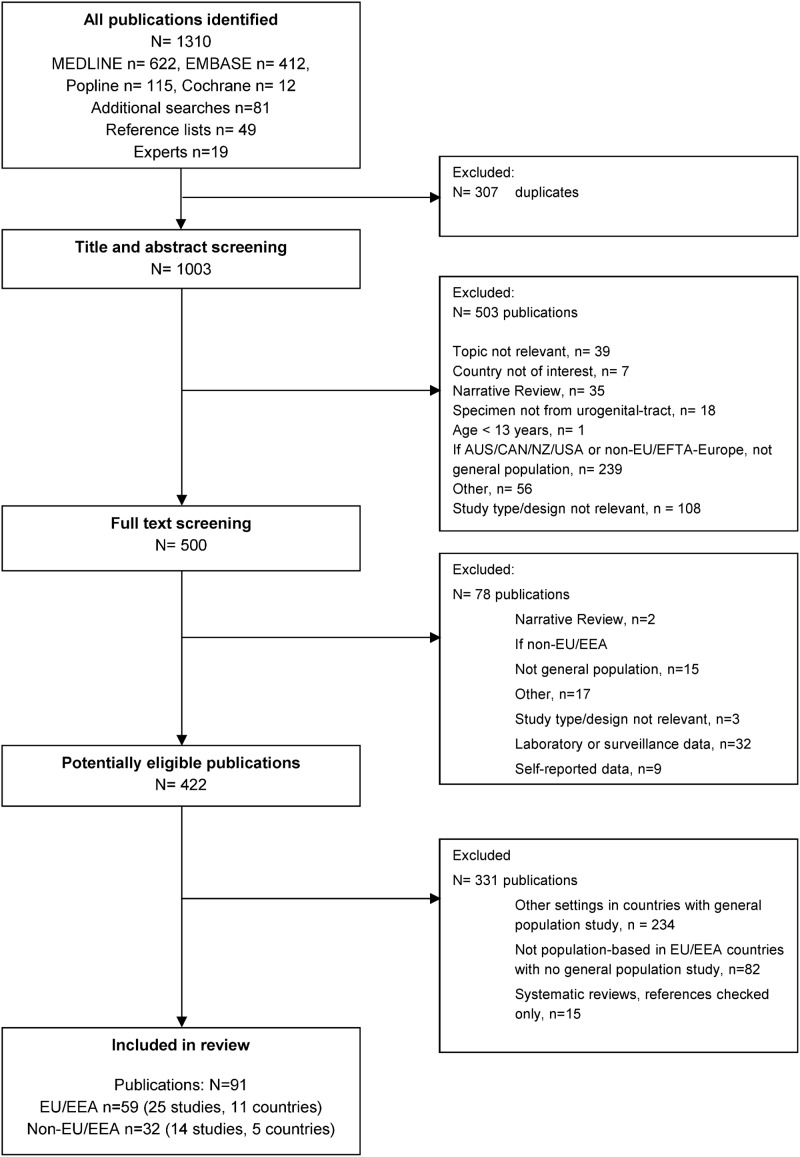
Flow diagram of study identification, inclusion and exclusion.

The search strategy gave a total of 1003 hits after de-duplication (Fig. 1). We included 25 primary studies (59 publications) in the populations of 11 EU/EEA countries [14, 16, 17, 19, 26–46] including Croatia, which became a Member State in July 2013 (Fig. 1) and 14 studies (32 publications) in five non-EU/EEA countries: Switzerland [47], Australia [48–51], Canada [52, 53], New Zealand [54] and the United States [18, 55–59]. We did not find any eligible studies from Israel, Japan or Korea. In the included studies, 121,915 (median 953, interquartile range 471 to 2,350) people in total were tested for chlamydia. Table 1 summarises the characteristics of each study.

**Table 1:** Summary of characteristics of included studies.

**Study name [ref.]**	**National or sub-national study**	**Sex, age in years**	**Whole study sample, sexually experienced only**	**Sample tested, test used**	**Number invited for testing (response rate overall, %)**	**Study name (acronym), if known; purpose of study, setting and sampling strategy**
**EU/EEA countries**						
Croatia 2011 [19]	National	W&M, 18–25	Sexually experienced only	Urine, NAAT	1005 participants 861 sexually experienced 280 provided urine sample (women 37.5%, men 27.9%)	Cross-sectional survey of sexual behaviour and STI prevalence. Nationally representative sample from all 21 counties in Croatia, with multi-stage probability sampling.
Denmark 1998 [26]	Sub-national	W&M, mean 18.0 women, 18.2 men	Sexually experienced only	Men first void urine, women urine and vaginal flush sample, NAAT	2603 women 928 eligible (women 33.3%) 1733 men 442 eligible (men 24.8%)	RCT of home sampling versus usual care. Random sample (half) of all high schools in Aarhus County. All students invited. Eligible if sexually experienced. (Only data from home sampling group included).
Denmark 1999 [27]	Sub-national	W, 20–29	Whole study sample	Cervical swab, NAAT	16345 eligible 11088 in cohort (women 67.8%)	Cohort study about risk factors for cervical cancer. Random sample of women born in Denmark, in catchment area of Righospitalet, Copenhagen taking part in a cohort study, who had cervical swab sample taken by gynaecologist.
Denmark 2001 [28]	Sub-national	M, 17–32	Both	Urine, NAAT	2500 (men 53.8%)	Cross-sectional survey to estimate chlamydia prevalence. All men in Northern Jutland, Aarhus or Copenhagen counties liable for military service and seen by a medical board.
Denmark 2002 [29]	Sub-national	W&M, 21–23	Sexually experienced only	Men first void urine, women vaginal flush sample, NAAT	4000 women (women 32.5% group 1,Response rates from online results for 26.3% group 2) 5000 men (men 25.9% group 1, 15.4% group 2)	RCT on effectiveness of outreach screening strategies. Simple random sample from all residents of Aarhus County in this age group. Group 1 received sampling kit, group 2 had to request kit by post.
Estonia 2008 [30]	Sub-national	W&M, 18–35	Whole study sample	Men urine, women vaginal swab, NAAT	1398 reachable (women 48%, men 32%)	Cross-sectional survey to estimate chlamydia prevalence. Stratified random sample of residents of Tartu county.
France 2010 [16]	National	W&M, 18–44	Sexually experienced only	Men urine, women vaginal swab (or urine), NAAT	4957 eligible by age and sexual experience (women 54.4%, men 49.3%)	Sexual behaviour survey (subsample of Contexte de la Sexualité en France study, NatChla). Random subsample of sexually experienced people from a national population-based survey on sexual behaviour with two-phase stratified sampling. Urine testing kit only sent to women if no swab returned after 1 month.
Germany 2012 [31]	National	W&M, 12–17	Both	Urine, NAAT	5755 in this age group (women and men 14–17 years 63%)	General health survey (Kinder und Jugendgesundheitsstudie, KiGGS). Two-stage stratified cluster sampling, nationally representative sample of 0–17 year olds. Only tested samples from participants in this age group.
Netherlands 2000 [32]	Sub-national	W&M, 15–40	Whole study sample	First void urine, NAAT	5714 women (women 50.8%) 5791 men (men 33.0%)	Cross-sectional survey to estimate chlamydia prevalence and screening feasibility. Simple random sample of patients on the lists of 16 general practices in Amsterdam.
Netherlands 2005 [33]	National	W&M, 15–29	Both	Urine, NAAT	20791 (women 47.0%, men 33.0%)	Cross-sectional survey to estimate chlamydia prevalence and screening feasibility (CT PILOT). Stratified probability sample of randomly selected men and women in 4 regions of the Netherlands according to population density. Regions not sampled at random.
Netherlands 2010 [34]	Sub-national	W&M, 16–29	Sexually experienced only	Men urine, women vaginal swab or urine, NAAT	140058 Amsterdam (women 22.4%, men 10.8%) 107806 Rotterdam (women 19.6%, men 10.5%)	Cluster controlled trial of chlamydia screening effectiveness (Chlamydia Screening Implementation, CSI). All 16–29 year old residents of Amsterdam, Rotterdam, parts of South Limburg. Sexually active people invited to request test kit. South Limburg excluded because eligibility depended on response to questionnaire assessing risk of chlamydia.
Norway 2005 [35]	Sub-national	W&M, 18–29	Whole study sample	Urine, NAAT	646 reached (women 43.8%, men 25%)	Cross-sectional survey to estimate chlamydia prevalence. All patients on the list of a group practice in Oslo.
Norway 2012 [36]	Sub-national	W&M, 18–25	Sexually experienced only	Urine, NAAT	10000 invited 1670 returned sample (women 18.9%, men 11.9%)	Cross-sectional survey to estimate chlamydia prevalence. Simple random sample of 10,000 people in this age group living in Rogaland county using unique personal identification number.
Slovenia 2004 [17]	National	W&M, 18–49	Both	First void urine, NAAT	2616 invited (women 60.0%, men 50.9%)	Sexual behaviour study. Stratified two stage probability sample of the general population of Slovenia in this age group. All participants invited to provide specimen for chlamydia testing.
Spain 2007 [37]	Sub-national	W, 15–44	Sexually experienced only	Cervical swab, NAAT	1821 invited 916 reached or accepted (women 66.1%)	Cross-sectional multinational HPV prevalence survey. Random age stratified sample of the adult female general population from census list of 4 urban communities in metropolitan Barcelona.
Sweden 1992 [38]	Sub-national	W, 15–34	Sexually experienced only	Cervical and urethral swabs, EIA (± direct IF)	543 reached and were sexually experienced women (68.9%)	Cross-sectional survey to estimate chlamydia prevalence. All women in this age group in a primary health care area in Nättraby invited, only sexually experienced screened.
Sweden 1995 [39]	Sub-national	W, 19, 21, 23, 25	Whole study sample	Cervical and urethral swabs, culture	816 reached 611 participated (68.3% women)	Cross-sectional survey to estimate chlamydia prevalence. All women of this age living in primary health care area of Ålidhem community centre in Umeå.
Sweden 2003 [40]	Sub-national	M, 22	Whole study sample	First void urine, NAAT	1074 (men 35.6%)	Cross-sectional survey to investigate feasibility of chlamydia screening. All males of this age living in Umeå.
Sweden 2004 [41]	Sub-national	W&M, 20–24	Whole study sample	First void urine, NAAT	200 (women 65%, men 45%)	Cross-sectional survey to estimate chlamydia prevalence and cost-effectiveness of home sampling. Simple random sample of 100 men and 100 women in this age group living in Umeå.
Sweden 2007 [42]	Sub-national	M, 19–24	Whole study sample	First void urine, NAAT	1936 reached (men 14.5%)	Cross-sectional survey to estimate chlamydia prevalence. Sampling method unclear, 1000 men living in Uppsala county (from population register), and 1000 Uppsala university students (from student register database).
United Kingdom 2000a [44]	Sub-national	M, 18–35	Whole study sample	First pass urine, NAAT	919 invited by post and reachable (men 45.3%)	Cross-sectional survey to estimate chlamydia prevalence and screening feasibility. Postal recruitment of all men aged 18–24 and a random sample of men aged 25–35 in 4 general practices in North West London.
United Kingdom 2000b [43]	Sub-national	W&M, 18–35	Sexually experienced only	Men urine, women urine or vulval swab, NAAT	166 women reached (women 39%) 175 men reached (men 46%)	Pilot study of acceptability of home sampling. Simple random sample of patients on the lists of 3 general practices in North West London and Avon. Urine samples from random 50% of women, vulval swabs from other 50%.
United Kingdom 2001 [14]	National	W&M, 18–44	Sexually experienced only	Urine, NAAT	5026 invited to give urine sample (women 71.1%, men 68.7%) ^a^	Sexual behaviour study (National Survey of Sexual Attitudes and Lifestyles, Natsal-2). Random sample of sexually experienced people taking part in a stratified probability sample of people aged 16–44 years resident in the United Kingdom (total 11 161 interviewed).
United Kingdom 2007 [45]	Sub-national	W&M, 16–39	Whole study sample	Men first void urine, women first void urine and vulvo-vaginal swab, NAAT	14382 reached (women 37.6%, men 27.9%)	Cross-sectional survey to estimate chlamydia prevalence and screening feasibility (Chlamydia Screening Studies project, ClaSS). Random sample of general population in Birmingham and Bristol areas, selected from 27 general practice lists.
United Kingdom 2012 [46]	Sub-national	W&M, 18–24	Whole study sample	Urine, NAAT	29917 invited (women 13.2%, men 9.8%)	Cross-sectional survey investigating feasibility of postal screening invitations. All people in this age group registered with any GP in North East Essex Primary Care Trust.
**Non-EU/EEA countries, Europe**						
Switzerland 2008 [47]	Sub-national	M, 18–26	Both	First void urine, NAAT	521 eligible and gave written consent (insufficient data to calculate)	Cross-sectional survey to estimate chlamydia prevalence. All young Swiss men attending obligatory medical board before army recruitment (French speaking region only).
**Non-EU/EEA countries, high income OECD**						
Australia 2003 [48]	Sub-national	W&M, 15–40+	Whole study sample	First catch urine, NAAT	6431 eligible 2862 participated (women and men 43.8%)	General health survey. All people living in 26 rural indigenous Australian and Torres Strait Islander communities in northern Queensland taking part in Well Person’s Health Check.
Australia 2004 [49]	Sub-national	W&M, 15–35	Whole study sample	Men first void urine, women vaginal swab, NAAT	2703 eligible listed 1219 screened (women 50.7%, men 39.3%)	Cross-sectional survey to estimate chlamydia and gonorrhoea prevalence. Indigenous Australian people aged 15–35 living in Alice Springs area
Australia 2006 [50]	Sub-national	W, 18–35	Both	First void urine, NAAT	1532 eligible households 979 women interviewed 657 gave urine sample (women 42.9%)	Cross-sectional survey to estimate chlamydia prevalence. Simple random sample from Melbourne residential telephone directory.
Australia 2008 [51]	Sub-national	W&M, 14–40	Whole study sample	Men first void urine, women low vaginal swabs, NAAT	ca. 1300 in 1996 (insufficient data to calculate)	Cross-sectional survey in STI control programme screening for chlamydia, gonorrhoea and syphilis. All resident indigenous Australians living in the Anangu Pitjantjatjara Yankunytjatjara Lands.
Canada 2002 [52]	Sub-national	W&M, 15–39	Whole study sample	First catch urine, NAAT	1075 women (women 29.3%) 1130 men (men 16.2%)	Chlamydia mass screening study. All adults from remote Inuit communities in Nunavik region. All sexually experienced or in this age group especially encouraged to take part.
Canada 2009 [53]	Sub-national	W&M, 15–65	Whole study sample	Urine, NAAT	224 estimated eligible (insufficient data to calculate) 181 screened (80.8% for women and men)	Chlamydia and gonorrhoea mass screening study. All men and women in this age group living in a rural Inuit community from Baffin Region, Nunavut.
New Zealand 2002 [54]	Sub-national	W&M, 16+	Sexually experienced only	Urine, NAAT	1582 invited 1136 consented 582 sexually active (insufficient data to calculate)	Cross-sectional survey to estimate chlamydia prevalence. Random sample of 50% of classes in all private and public high schools, Christchurch. Only sexually active had their samples tested.
USA 2001 [55]	Sub-national	W, 18–29	Sexually experienced only	Urine, NAAT	2148 eligible 1439 enrolled 1370 tested 1314 sexually active (women 61.2%)	Household survey of risk behaviour and chlamydia prevalence. All English- or Spanish-speaking women in this age group in a random sample of low income housing blocks from the 1990 census (<10^th^ percentile) in 3 counties in California.
USA 2002a [56]	National	M, 18–19, 22–26	Whole study sample	Urine, NAAT	1995 survey: data from 470 aged 18–19, and 995 aged 22–26 who were aged 15–19 in 1988 survey (insufficient data to calculate)	National Surveys of Adolescent Males (NSAM). Sexual health survey. Nationally representative sample of never-married, non-institutionalised men aged 15–19 (1995 survey), and aged 22–26 (aged 15–19 in 1988 survey but re-interviewed in 1995). Oversampling of black and Hispanic youths.
USA 2002b [57]	Sub-national	W&M, 18–35	Whole study sample	Urine, NAAT	1224 adults aged 18–45 reached 728 age-eligible for screening (women and men 79.5%)	Cross-sectional survey to estimate chlamydia and gonorrhoea prevalence. Stratified probability sampling of households in Baltimore; urine samples requested from those in study age group.
USA 2004 [58]	National	W&M, 18–26	Both	First void urine, NAAT	Wave III: 14322 (women and men 84%)	Cohort study (US National Longitudinal Study of Adolescent Health, Add Health). Nationally representative sample of young people in the USA. Total in first survey, Wave I: 18924.
USA 2011 [59]	Sub-national	W&M, 15–35	Both	Urine, NAAT	4998 eligible (women and men 42.7%)	Cross-sectional survey to estimate STI prevalence (Monitoring STI Survey Program). Probability sample of Baltimore residents.
USA 2012 [18]	National	W&M, 14–39	Whole study sample	Urine, NAAT	20836 selected 17190 interviewed (women 80.4%, 2007–2008, men 74.5%, 2007–2008) ^b^	General health survey (US National Health and Nutrition Examination Surveys, NHANES). Stratified multistage probability cluster sampling. Data from five 2-year survey cycles.

Abbreviations: EIA, enzyme immunoassay test; EU/EEA, European Union or European Economic Area Member States; IF, immunofluorescence test; M, men; NAAT, nucleic acid amplification test; OECD, Organization for Economic Cooperation and Development; STI, sexually transmitted infections; W: women.

^a^ Numbers from technical report Erens et al. 2001 [24].

^b^ Response rates from online results for 2007–2008 http://www.cdc.gov/nchs/nhanes/response_rates_CPS.htm.

Twenty seven studies included women and men [14, 16–19, 26, 29–36, 41, 43, 45, 46, 48, 49, 51–54, 57–59], six included only women [27, 37–39, 50, 55] and six included only men [28, 40, 42, 44, 47, 56]. The age group ranged from 15 to 17 years in a nationally representative survey in Germany [31] to 15 to 65 year olds in a single Arctic community in Canada [53]. Included studies ranged from nationally representative general health [18] or sexual lifestyle [14, 16, 17, 58] surveys to studies in localised populations, designed to test the feasibility of chlamydia screening interventions [43, 52, 54] or to get people tested and treated for chlamydia [42]. All but two studies [38, 39] used nucleic acid amplification tests (NAAT) for chlamydia diagnosis (Table 1) S1 Table lists the primary publication for each study and its associated publications.

### Risk of bias assessment

All included studies were at risk of biases that could affect the estimated chlamydia prevalence (S2 Table). The target population was assessed as being likely to be representative of the general population in only 8/39 studies; six studies in EU/EEA Member States Croatia [19], France [16], Germany [31], the Netherlands [33], Slovenia [17] and the UK [14] and two studies in the USA [18, 58]. Seventeen studies described a comparison between participants and non-participants. More than half of studies (23/39) did not give enough information about the source population to determine whether this was representative of the target population.

### Response rates

Authors of included studies used different denominators and numerators in their reported response rates. We calculated a response rate according to our algorithm for all but 4/39 studies [31, 41, 47, 51]. Amongst studies in EU/EEA countries, no study had a calculated response rate above 80%. The highest response rate (71%) was achieved as part of a national sexual behaviour survey in the UK [14]. Four studies had a response rate between 61% and 70% [27, 37–39]. The lowest response rates were in studies where entire populations in large geographic areas were invited by post; 13% in East Anglia, UK [46] and 16% in three regions in the Netherlands [34]. In non-EU countries, the calculated response rate was above 80% in two studies [53, 58], between 71% and 80% in two studies [18, 57] and between 61% and 70% in one study [55]. As with EU/EEA Member States, the highest response rates were obtained in studies of people who were already taking part in another study [18, 53, 57, 58].

### Chlamydia prevalence estimates

**Figure 2:**
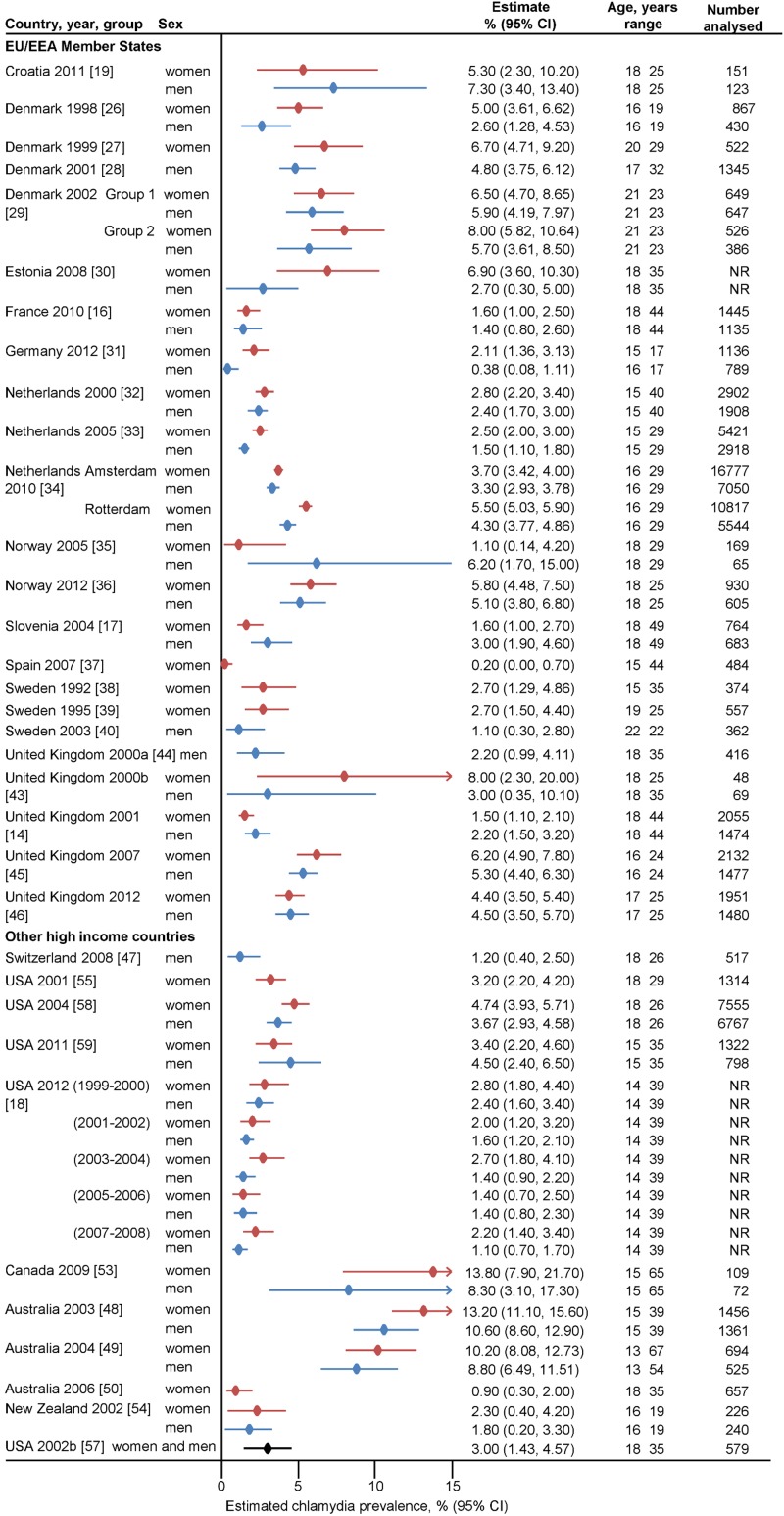
Forest plot, overall estimate of chlamydia prevalence in women and men of all ages in EU/EEA and other high-income OECD countries in all included studies. CI, confidence interval. The small filled diamond shows the point estimate, the lines either side are the 95% CI. Each row is a study or group within a study, with separate estimates from women and men, where available. In Denmark 2002, Group 1 received home sampling kits, Group 2 had to request a sampling kit by post. In USA 2012, separate estimates are reported for five survey cycles of the National Health and Nutrition Surveys. In Netherlands 2010, separate estimates were reported separately for Amsterdam and Rotterdam.

Fig. 2 shows the number of people included in the analysis and overall estimate of chlamydia prevalence for each included study. In EU/EEA countries, estimated prevalence in women ranged from 0.2% in sexually experienced 15 to 44 year olds in Barcelona, Spain in a study of human papillomavirus infection [37] to 8.0% in sexually experienced 21 to 23 year olds in Aarhus County, Denmark [29] and 18 to 25 year olds in London and Avon, UK [43], who were invited to take specimens at home in studies examining methods for chlamydia screening (Fig. 2). For men point prevalence estimates ranged from 0.4% amongst 16 to 17 year olds taking part in a general health survey in Germany [31] to 6.9% in sexually experienced male military recruits aged 17 to 32 years in three counties in Denmark [28] (Fig. 2). In the two studies that included only teenagers [26, 31], estimates were lower in men than in women (2.6% *vs.* 5.0% in Denmark, 0.4% *vs.* 2.1% in Germany). In non-EU/EEA countries, estimated prevalence in women ranged from 0.9% in 18 to 35 year olds in Melbourne, Australia [50] to 13.8% in a Canadian Arctic community aged 18 to 65 years [32] (Fig. 2). In men, the lowest estimated prevalences were in 14 to 39 year olds in a general health survey in the USA (1.1% [18]) and military recruits aged 18 to 26 years in the French-speaking region of Switzerland (1.2% [47]). The highest estimate was from 15 to 39 year olds in a remote community in Queensland, Australia (10.6% [48]).

**Figure 3:**
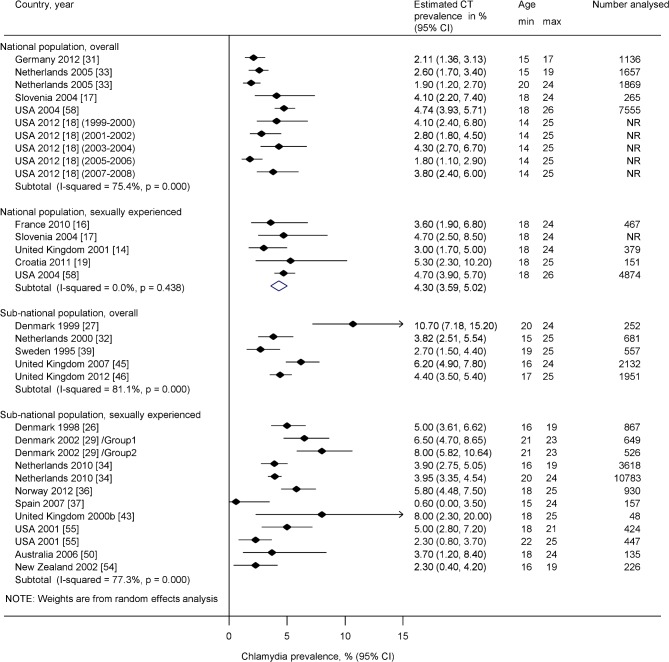
Forest plot, estimates of chlamydia prevalence in women ≤ 26 years in EU/EEA and other high-income OECD countries. CI, confidence interval. The small filled diamond shows the point estimate, the lines either side are the 95% CI. Each row is a study or group within a study. In Denmark 2002, Group 1 received home sampling kits, Group 2 had to request a sampling kit by post. Estimates are shown separately for sexually experienced participants only or for the overall sample, in either national or sub-national populations.

**Figure 4:**
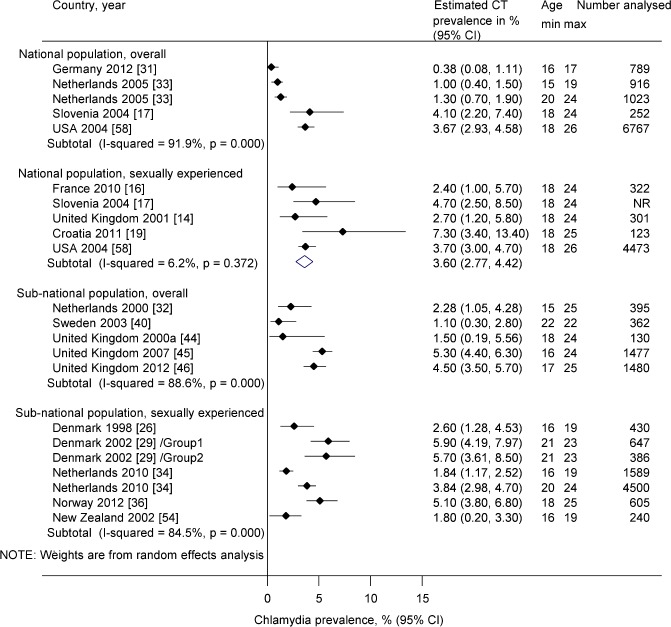
Forest plot, estimates of chlamydia prevalence in men ≤ 26 years in EU/EEA and other high-income OECD countries. CI, confidence interval. The small filled diamond shows the point estimate, the lines either side are the 95% CI. Each row is a study or group within a study. In Denmark 2002, Group 1 received home sampling kits, Group 2 had to request a sampling kit by post. Estimates are shown separately for sexually experienced participants only or for the overall sample, in either national or sub-national populations.

Fig. 3 and Fig. 4 show chlamydia prevalence estimates from studies conducted in EU/EEA and other high income OECD countries among women and men aged ≤26 years. In nationally representative samples of sexually experienced people in five countries, there was no or only mild heterogeneity. In women, estimates ranged from 3.0% (95% CI 1.7–5.0%) in the UK [14] to 5.3% (95% CI 2.3, 10.2%) in Croatia [19]. The pooled average estimate in all five countries was 4.3% (95% CI 3.6, 5.0%, I^2^ 0%) (Fig. 3) and in the four EU/EEA Member States 3.6% (95% CI 2.4, 4.8%, I^2^ 0%, not shown in the figure). In men, estimates ranged from 2.4% (95% CI 1.0, 5.7%) in France [16] to 7.3% (95% CI 3.4, 13.4%) in Croatia [19]. The pooled average estimate in all five countries was 3.6% (95% CI 2.8, 4.4%, I^2^ 6%) (Fig. 4) and in the four EU/EEA Member States 3.5% (95% CI 1.9, 5.2%, I^2^ 27%, not shown in the figure). Heterogeneity was severe (I^2^ >75%) in sub-national studies and in nationally representative studies with chlamydia prevalence estimates for the whole study population in both women and men; we did not estimate pooled averages for these groups of studies (Fig. 3 and Fig. 4).

**Figure 5:**
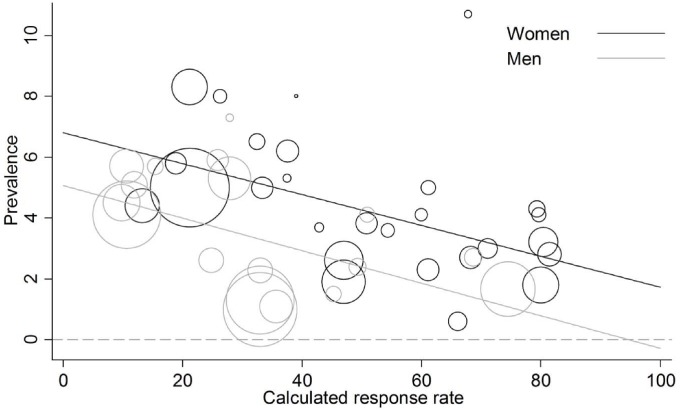
Meta-regression analysis of chlamydia prevalence estimates in women and men aged ≤25 years against calculated sex-specific response rate for all women and men in the study, in EU/EEA and other high-income OECD countries. The size of the open circle corresponds to the precision of the prevalence estimate. n = number of studies. For women, n = 27, P = 0.003, I^2^ 82.4%; men, n = 18, P = 0.018, I^2^ 87.6%.

**Figure 6:**
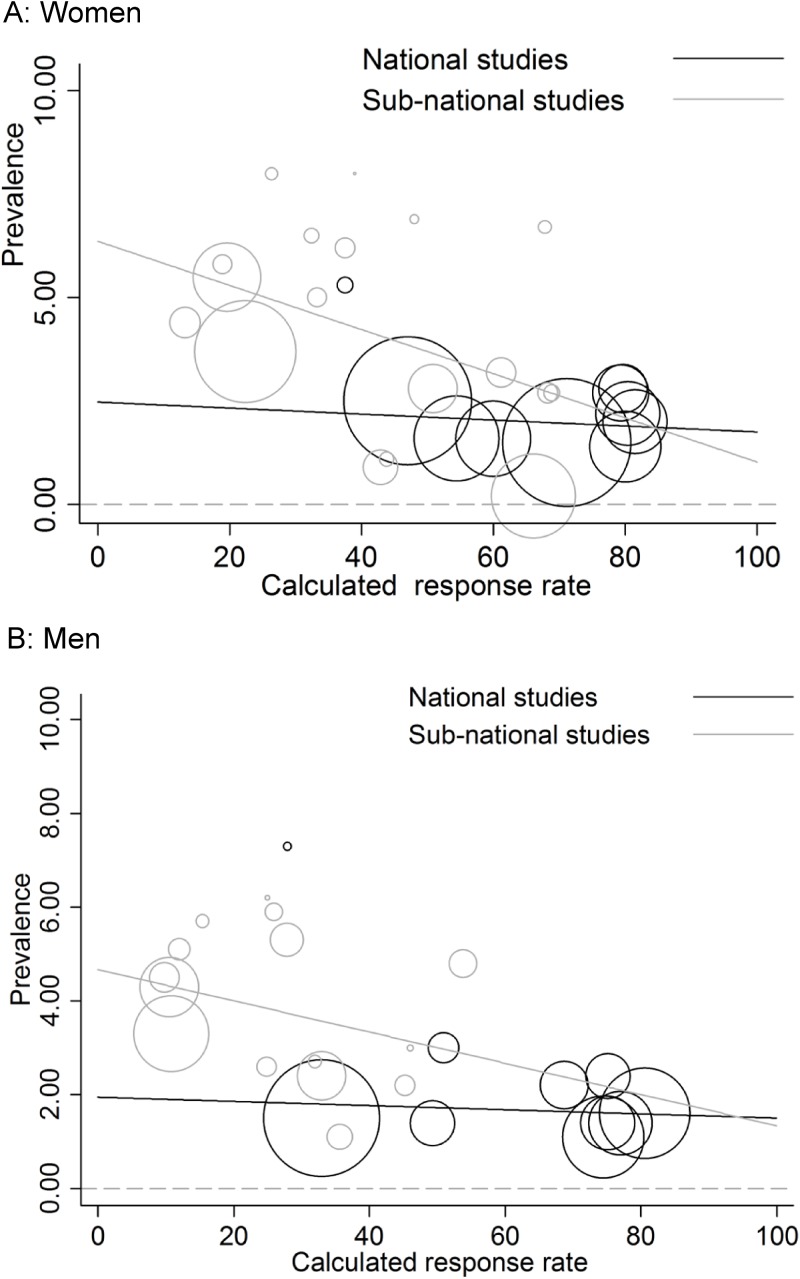
Meta-regression analysis of chlamydia prevalence estimates in participants of all ages against response rate, by national or sub-national study design. Panel A, women; Panel B, men. The size of the open circle corresponds to the precision of the prevalence estimate. n = number of studies. For women, national studies, n = 10, P = 0.644, I^2^ 46.8%; sub-national studies, n = 18 studies, P = 0.063, I^2^ 91.23%; for men, national studies, n = 10, P = 0.729, I^2^ 57.56%; sub-national studies, n = 15 studies, P = 0.267, I^2^ 81.25%.

There was statistical evidence of an association between overall sex-specific survey response rate and estimated chlamydia prevalence in both women and men; estimated chlamydia prevalence was higher in surveys with lower response rates (Fig. 5, women, P = 0.003; men, P = 0.018 from meta-regression). Results were similar if the analysis was restricted to studies that reported age-specific response rates for women and men aged ≤25 years (women, 15 studies, I^2^ 80.6%, P = 0.004; men, 13 studies, I^2^ 88.6%, P = 0.04). When the variable response rate was dichotomised (<60% and ≥60%), the ratio of odds for chlamydia infection was 1.9 times higher in studies with response rates <60% than in studies with response rates ≥60%. After controlling for national or sub-national study coverage, the ratio of odds was 1.7 (95% CI 0.9–3.2, P = 0.081). There was no strong evidence of an association between estimated chlamydia prevalence and response rate in surveys of nationally representative population samples in women (P = 0.644, Fig. 6A) or men (P = 0.729, Fig. 6B). In sub-national surveys, the meta-regression plot suggests an association between estimated chlamydia prevalence and with response rate (Fig. 6A and Fig. 6B). There was statistical evidence of this association in women (P = 0.063) but not men (P = 0.267) and there was substantial residual heterogeneity between prevalence estimates (I^2^ 91% women, 81% men). The regression lines for subnational and national surveys approached each other at higher levels of response rates. This suggests that at very high response rates, estimated prevalence would be similar in both survey types.

## Discussion

### Main findings

In this systematic review we found population-based surveys estimating chlamydia prevalence from 11 EU/EEA Member States, one non-EU/EEA European countries and four other high income countries. In nationally representative samples of sexually experienced ≤26 year olds, between study heterogeneity was low in women (five studies, range 3.0%, 95% CI 1.7, 5.0% in UK to 5.3%, 95% CI 2.3, 10.2% in Croatia, pooled estimate 4.3%, 95% CI 3.6, 5.0%, I^2^ 0%) and men (five studies, range 2.4%, 95% CI 1.0, 5.7% in France to 7.3%, 95% CI 3.4, 13.4% in Croatia, pooled estimate 3.6%, 95% CI 2.8, 4.4%, I^2^ 6.2%). Chlamydia prevalence estimates from population-based surveys conducted in sub-national population samples were very heterogeneous, ranging from 0.6% to 10.7% in women and 1.1% to 5.9% in men aged ≤25 years. Response rates in most included studies were <60%. There was statistical evidence of an inverse association between survey response rate and chlamydia prevalence estimates in both women (P = 0.003) and men (P = 0.018).

### Strengths and weaknesses of the review

Strengths of this review are the broad and inclusive search strategy and the detailed assessment of study methodology. We think that we are unlikely to have missed any large published articles, but might not have found all unpublished data. Our systematic searches covered studies published until August 2012. Since then, we identified one additional large survey of the UK population in 2010 to 2011 [60], which used methods similar to those of a survey from 1999 to 2000 [14]. Overall response rates and estimates of chlamydia prevalence were similar in both surveys. Another strength is that we only included studies that used population-based sampling methods to obtain estimates of chlamydia prevalence in the general population. Previous systematic reviews have included studies done in health care settings [1, 61, 62], the results of which cannot be easily extrapolated to the general population because they include people with symptoms and exposures that put them at higher than average risk of chlamydia infection. The inclusion of data from countries outside Europe increased statistical power to examine heterogeneity and allowed us to examine the generalisability of our findings to countries with similar levels of social and economic development. There was some inconsistency in the countries included in the review, however. Bulgaria, Hungary, Romania are EU Member States but not high-income economies; other high-income EU/EEA economies are not OECD members (Cyprus, Latvia, Liechtenstein, Lithuania, Malta). We did not find population-based studies in any of these countries. Two main limitations of the review relate to the small number of studies with comparable data and the completeness of the data reported. First, we could not calculate a consistent response rate for all studies because of differences between studies in the data reported and differences in study design. We overcame this limitation in part by applying an algorithm to select the numerator and denominator that were closest to the recommended definition [24]. The recommended numerator and denominator cannot be applied, however, in study designs that enrol participants and then restrict chlamydia testing to responders reporting sexual experience. In this case, the calculated response rate underestimates the true response rate and cannot be corrected unless the percentages excluded because they have not had sexual experience are recorded. Second, four countries (Denmark, Netherlands, Sweden, UK) accounted 17/25 included studies from EU/EEA countries. The small number of countries contributing to the review needs to be considered when interpreting the findings.

### Interpretation

Estimates of chlamydia prevalence in women and men aged ≤26 years in surveys of nationally representative samples of populations in EU/EEA and other high income countries were statistically consistent and between study variability was compatible with random variation [23]. The pooled estimates for EU/EEA Member States are the average of estimates of chlamydia prevalence from four studies and do not mean that this is the chlamydia prevalence across Europe. The chlamydia prevalence estimates and their precision need to be interpreted in the context of national differences in culture, sexual behaviours and attitudes, health systems and intensity and duration of chlamydia control activities [63, 64]. Most of the point estimates of chlamydia prevalence were <5% in both women and men. Participation bias might still affect these estimates because of low response rates and the low estimated prevalence of chlamydia [12]. Over-estimation is more likely than under-estimation because responders have higher levels of factors associated with STI than non-responders [14].

In cross-sectional surveys of chlamydia prevalence, the lower the calculated response rate the higher was the estimated prevalence. The association appeared to be more marked in studies conducted in sub-national regions of a country than in nationally representative population surveys (Fig. 6). Differences in the objectives of studies in these groups could help explain this finding. The objectives of sub-national studies were diverse. Studies that assessed the feasibility of chlamydia screening approaches might have specifically encouraged chlamydia testing by people at high risk of infection but have low overall response rates [29, 34, 45]. Studies designed to measure chlamydia prevalence as a main [50] or subsidiary objective [37] might have enrolled a more representative sample of the target population. In nationally representative surveys, chlamydia testing was done as a small part of studies that were designed to measure a wide range of health-related [58] or sexual health-related behaviours [14, 16, 17]. These studies tended to have higher overall response rates than sub-national studies. Of note, the national survey with the highest estimate of chlamydia prevalence, in Croatia, also had the lowest response rate [19].

### Implications for practice, policy and research

This review highlights several challenges to determining accurate and comparable estimates of chlamydia prevalence between countries. Standard definitions used by survey and market research organisations to define target and study populations and to calculate response rates were rarely adhered to. Reporting standards for prevalence surveys in epidemiological research, perhaps as an extension to existing Standards for the Reporting of Observational Studies in Epidemiology [65] might help to improve consistency in future. The association between estimated chlamydia prevalence and survey response rate suggests that estimates from studies with very low response rates should not be interpreted as estimates of the population chlamydia prevalence, even when sampling has covered a whole defined region of a country. This review does not provide data to specify a threshold response rate below which the value estimated is unreliable, however. Our review shows that population-based chlamydia prevalence has been estimated in a minority of European and other high income countries. Surveys among samples representative of national populations in a wider variety of countries, particularly in non-high income EU Member States, and in other low and middle income countries would be valuable if they use consistent methods and achieve high response rates. Surveys that estimate chlamydia prevalence are at risk of participation bias owing to low response rates; estimates obtained in nationally representative samples of the general population of EU/EEA Member States are similar to estimates from other high income countries.

## Supporting Information

S1 PRISMA Checklist PRISMA checklist. (PDF)

S1 Table Bibliography of primary and associated publications, by region and country in alphabetical order. (PDF)

S2 Table Assessment of risk of bias in included studies. (PDF)

S1 Text Protocol for the systematic review. (PDF)
