# Supplementary material for: Genital Chlamydia Prevalence in Europe and Non-European High Income Countries: Systematic Review and Meta-Analysis
Source: PLoS One. 2015 Jan 23;10(1):e0115753. doi: 10.1371/journal.pone.0115753 (PMC4304822; doi:10.1371/journal.pone.0115753)
Supplement: S1 Table — (PDF) [file pone.0115753.s002.pdf]

Table S1

Bibliography of primary and associated publications, by region and country in alphabetical order

Study name, reference	Main publication	Additional publications
Croatia 2011 [19]	Bozicevic I, Grgic I, Zidovec-Lepej S, Cakalo JI, Belak-Kovacevic S, Stulhofer A et al. (2011) Urine-based testing for Chlamydia trachomatis among young adults in a population-based survey in Croatia: feasibility and prevalence. BMC Public Health 11: 230. 10.1186/1471-2458-11-230 [doi]..	
Denmark 1998 [26]	Ostergaard L, Andersen B, Olesen F, Moller JK (1998) Efficacy of home sampling for screening of Chlamydia trachomatis: randomised study. BMJ 317(7150): 26-27.	Ostergaard L, Andersen B, Moller JK, Olesen F (2000) Home sampling versus conventional swab sampling for screening of chlamydia trachomatis in women: A cluster-randomized 1-year follow-up study. Clin Infect Dis 31(4): 951-957.
Denmark 1999 [27]	Munk C, Morre SA, Kjaer SK, Poll PA, Bock JE, Meijer, CJ et al. (1999) PCR-detected Chlamydia trachomatis infections from the uterine cervix of young women from the general population: prevalence and risk determinants. Sex Transm Dis 26(6): 325-328.	Kjaer SK, van den Brule AJ, Bock JE, Poll PA, Engholm G, Sherman ME et al. (1996) Human papillomavirus--the most significant risk determinant of cervical intraepithelial neoplasia. Int J Cancer 65(5): 601-606.
Denmark 2001 [28]	Bennedsen M, Nygard B, Berthelsen L, Jensen JS, Lind I (2001) [Prevalence of Chlamydia among young men. A screening among men liable for military service and coming before the military board]. Ugeskr Laeger 163(34): 4583-6.	
Denmark 2002 [29]	Andersen B, Olesen F, Moller JK, Ostergaard L (2002) Population-Based Strategies for Outreach Screening of Urogenital Chlamydia trachomatis Infections: A Randomized, Controlled Trial. J Infect Dis 185(2): 252-258.	Moller JK, Andersen B, Olesen F, Lignell T, Ostergaard L (1999) Impact of menstrual cycle on the diagnostic performance of LCR, TMA, and PCE for detection of Chlamydia trachomatis in home obtained and mailed vaginal flush and urine samples. Sex Transm Infect 75(4):228-230.
		Andersen B, van Valkengoed I, Sokolowski I, Moller JK, Ostergaard L, Olesen F (2011) Impact of intensified testing for urogenital Chlamydia trachomatis infections: a randomised study with 9-year follow-up. Sex Transm Infect 87(2): 156-161.
Estonia 2008 [30]	Uuskula A, Kals M, Denks K, Nurm UK, Kasesalu L, Dehovitz J et al.(2008) The prevalence of chlamydial infection in Estonia: a population-based survey. International Journal of STD AIDS 19(7): 455-458.	Uuskula A, Kals M, McNutt L-A (2011) Assessing non-response to a mailed health survey including self-collection of biological material. European Journal of Public Health 21(4):538-542
France 2010 [16]	Goulet V, de Barbeyrac B, Raherison S, Prudhomme M, Semaille C, Warszawski J (2010) Prevalence of Chlamydia trachomatis: results from the first national population-based survey in France. Sex Transm Infect 86 (4): 263-270.	Bajos N, Bozon M, Beltzer N, Laborde C, Andro A, Ferrand M et al. (2010) Changes in sexual behaviours: from secular trends to public health policies. AIDS 24(8):1185-1191
		Goulet, V, De Barbeyrac B, Raherison S, Prudhomme M, Velter A, Semaille C et al. (2011). "National survey on Chlamydia trachomatis infection in France (NatChla Study, CSF 2006 Survey). To whom should screening be proposed? [Enquête nationale de prévalence de l'infection à Chlamydia trachomatis (volet NatChla de l'enquête CSF 2006). À quelles personnes proposer un dépistage ?]." Bulletin épidémiologique hebdomadaire 12(5 avril 2011): 160-164.
Germany 2012	Haar K, (2012) "Prävalenz von urogenitalen Chlamydia trachomatis-	Haar K, Bremer V, Houareau C, Meyer T, Desai S, Thamm M, et al. (2013) Risk factors for Chlamydia trachomatis infection in adolescents: results from a

[31]	Infektionen bei Teilnehmern des bundesweiten Kinder- und Jugendgesunheitssurveys (KiGGS)“Masterarbeit für MPH, Berlin School of Publich Health, Robert Koch Institute, Berlin.	representative population-based survey in Germany, 2003-2006. Euro Surveill. 18 (34): pii=20562<br><br>Desai S, Meyer T, Thamm M, Hamouda O, Bermer V (2011). "Prevalence of Chlamydia trachomatis among young German adolescents, 2005-06." Sexual Health 8(1): 120-122.<br><br>Kurth B-M, Kamtsiuris P, Holling H, Schlaud M, Dolle R, Ellert U et al. (2008). "The challenge of comprehensively mapping children's health in a nation-wide health survey: Design of the German KiGGS-Study." BMC Public Health 8(1): 196.<br><br>Thierfelder W, Dortschy R, Hintzpeter B, Kahl H, Scheidt-Nave C (2007). "[Biochemical measures in the German Health Interview and Examination Survey for Children and Adolescents (KiGGS)]." Bundesgesundheits-blatt Gesundheitsforschung Gesundheitsschutz 50(5-6): 757-770.<br><br>Kamtsiuris P, Lange M, Schaffrath RA (2007). "[The German Health Interview and Examinations Survey for Children and Adolescents (KiGGS): Sample design, response and nonresponse analysis]." Bundesgesundheitsblatt Gesundheitsforschung Gesundheitsschutz 50(5-6): 547-556.
Netherlands 2000 [32]	van Valkengoed I G, Morre SA, van den Brule AJ, Meijer CJ, Deville W, Bouter LM et al. (2000). "Low diagnostic accuracy of selective screening criteria for asymptomatic Chlamydia trachomatis infections in the general population." Sexually Transmitted Infections 76(5): 375-380.	van Valkengoed IG, Boeke AJ, Morre SA, van den Brule AJ, Meijer CJ, Deville W et al. (2000) Disappointing performance of literature-derived selective screening criteria for asymptomatic Chlamydia trachomatis infection in an inner-city population. Sex Transm Dis 27(9): 504-507.<br><br>van Valkengoed GM, Boeke AJP, van den Brule AJC, Morre SA, Dekker JH, Meijer CJLM et al. (1999) Systematic screening for asymptomatic Chlamydia trachomatis infections by home obtained mailed urine samples in men and women in general practice. Nederlands Tijdschrift voor Geneeskunde 143(13): 672-676.
Netherlands 2005 [33]	van Bergen J, Gotz HM, Richardus JH, Hoebe CJ, Broer J, Coenen AJ (2005) Prevalence of urogenital Chlamydia trachomatis increases significantly with level of urbanisation and suggests targeted screening approaches: results from the first national population based study in the Netherlands. Sex Transm Infect 81(1): 17-23.	van Bergen JEAM, Gotz HM, Richardus JH, Hoebe CJPA, Broer J, Coenen AJJ (2005) Chlamydia trachomatis infection in 4 regions in the Netherlands: Results of a population-based study conducted through municipal health services and implications for screening. Nederlands Tijdschrift voor Geneeskunde 149(39):2167-2174<br><br>Veldhuijzen IK, van Bergen JEAM, Gotz HM, Hoebe CJPA, Morre SA, Richardus JH, Pilot Ct Study Group (2005) Reinfections, persistent infections, and new infections after general population screening for Chlamydia trachomatis infection in the Netherlands. Sex Transm Dis 32(10): 599-604.<br><br>Gotz HM, van Bergen JE, Veldhuijzen IK, Broer J, Hoebe CJ, Steyerberg EW et al. (2005) A prediction rule for selective screening of Chlamydia trachomatis infection. Sex Transm Infect 81(1): 24-30.<br><br>Gotz HM, Hoebe CJ, van Bergen JE, Veldhuijzen IK, Broer J, de Groot F et al. (2005) Management of Chlamydia cases and their partners: results from a home-based screening program organized by municipal public health services with referral to regular health care. Sex Transm Dis 32(10): 625-629.<br><br>Gotz HM, Veldhuijzen IK, van Bergen JE, Hoebe CJ de Zwatt O, Richardus JH et al. (2005). "Acceptability and consequences of screening for chlamydia trachomatis by home-based urine testing." Sex Transm Dis 32(9): 557-562.<br><br>Gotz HM, van Bergen JEAM, Veldhuijzen IK, Hoebe CJPA, Broer J, Coenen AJJ et al. (2006) Lessons learned from a population-based chlamydia screening pilot.

		International Journal of STD & AIDS 17(12): 826-830.<br><br>Gotz HM, Veldhuijzen IK, Habbema JD, Boeke AJ, Richardus JH, Steyerberg EW (2006) Prediction of Chlamydia trachomatis infection: application of a scoring rule to other populations. Sex Transm Dis 33(6): 374-380.<br><br>van Bergen J, Gotz H, Richardus JH, Hoebe C, Broer J, Coenen T, Pilot Ct Study Group (2006) Prevalence of urogenital Chlamydia trachomatis infections in the Netherlands suggests selective screening approaches. Results from the PILOT CT Population Study. Drugs of Today 42 Suppl A: 25-33.
Netherlands 2010 [34]	van Bergen JE, Fennema JS, van den Broek IV, Brouwers EE, de Feijter EM, Hoebe CJ et al. (2010) Rationale, design, and results of the first screening round of a comprehensive, register-based, Chlamydia screening implementation programme in the Netherlands. BMC Infect Dis 10: 293. 10.1186/1471-2334-10-293 [doi].	van den Broek IVF, Hoebe CJPA, van Bergen JEAM, Brouwers EEHG, de Feijter EM, Fennema JSA et al. (2010) Evaluation design of a systematic, selective, internet-based, Chlamydia Screening Implementation in the Netherlands, 2008-2010: implications of first results for the analysis. BMC Infect Dis 10: 89.<br><br>Greenland KE, Op de Coul EL, van Bergen JE, Brouwers EE, Fennema HJ, Gotz HM et al. (2011) Acceptability of the Internet-Based Chlamydia Screening Implementation in the Netherlands and Insights Into Nonresponse. Sex Transm Dis . 38(6): 467-474.<br><br>Op de Coul ELM, Gotz HM, van Bergen JEAM, Fennema JSA, Hoebe CJPA, Koekenbier RH et al. (2012) Who Participates in the Dutch Chlamydia Screening? A Study on Demographic and Behavioral Correlates of Participation and Positivity. Sex Transm Dis 39(2): 97-103.<br><br>van den Broek IV, van Bergen JE, Brouwers EE, Fennema HJ, Gotz HM, Hoebe CJ et al. (2012) Effectiveness of yearly, register based screening for chlamydia in the Netherlands: controlled trial with randomised stepped wedge implementation. BMJ 345: e4316. doi: 10.1136/bmj.e4316<br><br>Gotz HM, van den Broek IV, Hoebe CJ, Brouwers EE, Pars LL, Fennema JS et al. (2012). "High yield of reinfections by home-based automatic rescreening of Chlamydia positives in a large-scale register-based screening programme and determinants of repeat infections." Sex Transm Infect.10.1136/sextrans-2011-050455 doi
Norway 2005 [35]	Steen TW, Hjortdahl P, Storvold G, Vilimas K, Elstrom P, Esholdt I et al. (2005) [Prevalence of genital Chlamydia trachomatis infection in the age group 18-29 years in Oslo]. Tidsskrift for Den Norske Laegeforening 125(2): 1637-9	
Norway 2012 [36]	Klovstad H, Grjibovski A, Aavitsland P (2012) Population based study of genital Chlamydia trachomatis prevalence and associated factors in Norway: A cross sectional study. BMC Infect Dis 12(1):150 doi:10.1186/1471-2334-12-150	
Slovenia 2004 [17]	Klavs I, Rodrigues LC, Wellings K, Kese D, Hayes R (2004) Prevalence of genital Chlamydia trachomatis infection in the general population of Slovenia: serious gaps in control. Sex Transm Infect 80(2): 121-123.	Klavs I, Rodrigues LC, Wellings K, Kese D, Svab I (2002) Feasibility of testing for Chlamydia trachomatis in a general population sexual behaviour survey in Slovenia. International Journal of STD & AIDS 13 Suppl 2: 5-8.
Spain 2007 [37]	Franceschi S, Smith JS, van den Brule A, Herrero R, Arslan A, Anh P-T-H et al. (2007) Cervical infection with Chlamydia trachomatis and Neisseria gonorrhoeae in women from ten areas in four	de Sanjose S, Almirall R, Lloveras B, Font R, Diaz M, Muñoz N et al. (2003) Cervical Human Papillomavirus Infection in the Female Population in Barcelona, Spain. Sex Transm Dis 30(10): 788-793

	continents. A cross-sectional study. Sex Transm Dis 34(8): 563-9	
Sweden 1992 [38]	Brannstrom M, Josefsson GB, Cederberg A, Liljestrand J (1992) Prevalence of genital Chlamydia trachomatis infection among women in a Swedish primary health care area. Scandinavian Journal of Infectious Diseases 24(1): 41-6.	
Sweden 1995 [39]	Jonsson M, Karlsson R, Rylander E, Boden E, Edlund K, Evander M et al.(1995) The silent suffering women--a population based study on the association between reported symptoms and past and present infections of the lower genital tract. Genitourin Med 71(3): 158-162.	Jonsson M, Karlsson R, Persson K, Juto P, Edlund K, Evander M et al.(1995) The influence of sexual and social factors on the risk of Chlamydia trachomatis infections: A population-based serologic study. Sex Transm Dis 22(6): 355-363.<br><br>Karlsson R, Jonsson M, Edlund K, Evander M, Gustavsson A, Boden E et al. (1995) Lifetime number of partners as the only independent risk factor for human papillomavirus infection: a population-based study. Sex Transm Dis 22(2): 119-27
Sweden 2003 [40]	Novak DP, Edman AC, Jonsson M, Karlsson RB (2003) The internet, a simple and convenient tool in Chlamydia trachomatis screening of young people. Euro Surveillance: European Communicable Disease Bulletin 8(9): 171-176.	
Sweden 2004 [41]	Novak DP, Lindholm L, Jonsson M, Karlsson RB (2004) A Swedish cost-effectiveness analysis of community-based Chlamydia trachomatis PCR testing of postal urine specimens obtained at home. Scandinavian Journal of Public Health 32(5):324-32	
Sweden 2007 [42]	Domeika M, Oscarsson L, Hallen A, Hjelm E, Sylvan S (2007) Mailed urine samples are not an effective screening approach for Chlamydia trachomatis case finding among young men. Journal of the European Academy of Dermatology & Venereology 21(6):789-94	
United Kingdom 2000a [43]	Stephenson J, Carder C, Copas A, Robinson A, Ridgway G, Haines A (2000) Home screening for chlamydial genital infection: is it acceptable to young men and women? Sex Transm Infect 76(1): 25-27.	
United Kingdom 2000b [44]	Pierpoint T, Thomas B, Judd A, Brugha R, Taylor-Robinson D, Renton A (2000) Prevalence of Chlamydia trachomatis in young men in north west London. Sex Transm Infect 76(4): 273-276.	
United Kingdom 2001 [14]	Fenton KA, Korovessis C, Johnson AM, McCadden A, McManus S, Wellings K et al. (2001) Sexual behaviour in Britain: reported sexually transmitted infections and prevalent genital *Chlamydia trachomatis* infection. Lancet 358(9296): 1851-1854.	Johnson AM, Mercer CH, Erens B, Copas AJ, McManus S, Wellings K et al. (2001) Sexual behaviour in Britain: partnerships, practices and HIV risk behaviours. Lancet 358 (9296): 1835-1842.<br><br>McCadden A, Fenton KA, McManus S, Mercer CH, Erens B, Carder C et al. (2005) Chlamydia trachomatis testing in the second British national survey of sexual attitudes and lifestyles: respondent uptake and treatment outcomes. Sex Transm Dis 32(6):387-394.
United Kingdom 2007 [45]	Low N, McCarthy A, Macleod J, Salisbury C, Campbell R, Roberts TE et al. (2007) Epidemiological, social, diagnostic and economic evaluation of population	Low N, McCarthy A, Macleod J, Salisbury C, Horner PJ, Roberts TE et al. for the Chlamydia Screening Studies Group (2004) The chlamydia screening studies: rationale and design. Sex Transm Infect 80(5): 342-348.

	screening for genital chlamydial infection. Health Technol Assess 11: 1-184.	Macleod J, Salisbury C, Low N, McCarthy A, Sterne JA, Holloway A et al. (2005) Coverage and uptake of systematic postal screening for genital Chlamydia trachomatis and prevalence of infection in the United Kingdom general population: cross sectional study. BMJ 330(7497): 940-942.
United Kingdom 2012 [46]	Bracebridge S, Bachmann MO, Ramkhelawon K, Woolnough A (2012) Evaluation of a systematic postal screening and treatment service for genital Chlamydia trachomatis, with remote clinic access via the internet: a cross-sectional study, East of England. Sex Transm Infect .	
**Non-EU/EEA countries, Europe**		
Switzerland 2008 [47]	Baud D, Jaton K, Bertelli C, Kulling JP, Greub G (2008) Low prevalence of Chlamydia trachomatis infection in asymptomatic young Swiss men. BMC Infect Dis 8: 45.	
**Non-EU/EEA countries, high income OECD**		
Australia 2003 [48]	Miller GC, McDermott R, McCulloch B, Fairley CK, Muller R (2003) Predictors of the prevalence of bacterial STI among young disadvantaged Indigenous people in north Queensland, Australia. Sex Transm Infect 79:332-335.	Miller PJ, Torzillo PJ, Hateley W (1999) Impact of improved diagnosis and treatment on prevalence of gonorrhoea and chlamydial infection in remote aboriginal communities on Anangu Pitjantjatjara Lands. Medical Journal of Australia 170(9): 429-432.<br><br>Miller G, McDermott R, McCulloch B, Leonard D, Arabena K, Muller R (2002) The Well Person's Health Check: a population screening program in indigenous communities in north Queensland. Australian Health Review 25(6): 136-147.
Australia 2004 [49]	Latif A, Smith K (2004) STI screening conducted in NT Department of Health and Community Services and Community Controlled Health Services in Central Australia in 2004. The Northern Territory Disease Control Bulletin 11(4): 18-20.	
Australia 2006 [50]	Hocking JS, Willis J, Tabrizi S, Fairley CK, Garland SM, Hellard M (2006) A chlamydia prevalence survey of young women living in Melbourne, Victoria. Sexual Health 3(4): 235-240.	
Australia 2008 [51]	Huang R-L, Torzillo PJ, Hammond VA, Coulter ST, Kirby AC (2008) Epidemiology of sexually transmitted infections on the Anangu Pitjantjatjara Yankunytjatjara Lands: results of a comprehensive control program. Medical Journal of Australia 189(8): 442-445.	
Canada 2002 [52]	Hodgins S, Peeling RW, Dery S, Bernier F, LaBrecque A, Proulx JF et al.(2002) The value of mass screening for chlamydia control in high prevalence communities. Sex Transm Infect 78(Suppl 1): i64-i68.	
Canada 2009 [53]	Steenbeek A, Tyndall M, Sheps S, Rothenberg R (2009) An epidemiological survey of chlamydial and gonococcal infections in a Canadian arctic community. Sex Transm Dis 36(2): 79-83.	
New Zealand 2002 [54]	Corwin P, Abel G, Wells JE, Coughlan E, Bagshaw S, Sutherland M et al. (2002) Chlamydia trachomatis prevalence and	Abel G, Brunton C (2005) Young people's use of condoms and their perceived vulnerability to sexually transmitted infections. Australian and New Zealand Journal of Public Health 29(3): 254-260.

	sexual behaviour in Christchurch high school students. New Zealand Medical Journal 115(1158):U107.	
USA 2001 [55]	Klausner JD, McFarland W, Bolan G, Hernandez MT, Molitor F, Lemp GF et al. (2001) Knock-Knock: A Population-Based Survey of Risk Behavior, Health Care Access, and Chlamydia trachomatis Infection among Low-Income Women in the San Francisco Bay Area. J Infect Dis 183(7): 1087-1092.	Ruiz JD, Molitor F, McFarland W, Klausner J, Lemp G, Page-Shafer K et al. (2000) Prevalence of HIV infection, sexually transmitted diseases, and hepatitis and related risk behavior in young women living in low-income neighborhoods of northern California. West J Med 172(6):368-373.
USA 2002a [56]	Ku L, St.Louis M, Farshy C, Aral S, Turner CF, Lindberg LD, Sonenstein F (2002) Risk Behaviors, Medical Care, and Chlamydial Infection Among Young Men in the United States. Am J Public Health 92(7): 1140-1143.	
USA 2002b [57]	Turner CF, Rogers SM, Miller HG, Miller WC, Gribble JN, Chromy JR et al. (2002) Untreated gonococcal and chlamydial infection in a probability sample of adults. JAMA 287(6): 726-733.	
USA 2004 [58]	Miller WC, Ford CA, Morris M, Handcock MS, Schmitz JL, Hobbs MM et al. (2004) Prevalence of chlamydial and gonococcal infections among young adults in the United States. JAMA 291(18): 2229-2236.	Ford CA, Jaccard J, Millstein SG, Bardsley PE, Miller WC (2004) Perceived risk of chlamydial and gonococcal infection among sexually experienced young adults in the United States. Perspectives on Sexual & Reproductive Health 36(6): 258-264.
		Iritani BJ, Ford CA, Miller WC, Hallfors DD, Halpern CT (2006) Comparison of self-reported and test-identified chlamydial infections among young adults in the United States of America. Sexual Health 3(4): 245-251.
		Geisler WM, Chyu L, Kusunoki Y, Upchurch DM, Hook EW, III (2006) Health insurance coverage, health care-seeking behaviors, and genital chlamydial infection prevalence in sexually active young adults. Sex Transm Dis 33(6): 389-396.
		Manhart LE, Holmes KK, Hughes JP, Houston LS, Totten PA (2007) Mycoplasma genitalium among young adults in the United States: An emerging sexually transmitted infection. Am J Public Health 97(6): 1118-1125.
		Stein CR, Kaufman JS, Ford CA, Leone PA, Feldblum PJ, Miller WC (2008) Screening young adults for prevalent chlamydial infection in community settings. Ann Epidemiol 18(7): 560-571.
		Stein CR, Kaufman JS, Ford CA, Feldblum PJ, Leone PA, Miller WC (2008) Partner age difference and prevalence of chlamydial infection among young adult women. Sex Transm Dis 35(5): 447-452.
		Annang L, Walsemann KM, Maitra D, Kerr JC (2010) Does education matter? Examining racial differences in the association between education and STI diagnosis among black and white young adult females in the U.S. Public Health Reports 125 Suppl 4: 110-121
USA 2011 [59]	Eggleston E, Rogers SM, Turner CF, Miller WC, Roman AM, Hobbs MM et al. (2011) Chlamydia trachomatis infection among 15-to 35-year-olds in Baltimore, MD. Sex Transm Dis 38(8): 743-749.	Eggleston E, Turner CF, Rogers SM, Roman A, Miller WC, Villarroel MA et al.(2005) Monitoring STI prevalence using telephone surveys and mailed urine specimens: A pilot test. Sex Transm Infect 81(3): 236-238.

USA 2012 [18]	Datta SD, Torrone E, Kruszon-Moran D, Berman S, Johnson R, Satterwhite CL et al. (2012) Chlamydia trachomatis Trends in the United States Among Persons 14 to 39 Years of Age, 1999-2008. Sex Transm Dis 39(2): 92-96.	Satterwhite C L, Torrone E, Meites E, Dunne EF, Mahajan R, Ocfemia MC et al. (2013). Sexually transmitted infections among US women and men: prevalence and incidence estimates, 2008. Sex Transm Dis 40(3): 187-193.<br><br>Datta SD, Sternberg M, Johnson RE, Berman S, Papp JR, McQuillan G et al. (2007) Gonorrhea and Chlamydia in the United States among Persons 14 to 39 Years of Age, 1999 to 2002. Ann Intern Med 147(2): 89-96.<br><br>Forhan SE, Gottlieb SL, Sternberg MR, Xu F, Datta SD, McQuillan GM et al. (2009) Prevalence of sexually transmitted infections among female adolescents aged 14 to 19 in the United States. Pediatrics 124(6): 1505-1512.<br><br>Allsworth JE, Ratner JA, Peipert JF (2009) Trichomoniasis and other sexually transmitted infections: Results from the 2001-2004 national health and nutrition examination surveys. Sex Transm Dis 36(12): 738-744.<br><br>Beydoun HA, Dail J, Tamim H, Ugwu B, Beydoun MA (2010) Gender and age disparities in the prevalence of chlamydia infection among sexually active adults in the United States. Journal of Women's Health 19(12): 2183-2190.<br><br>Centers for Disease Control and Prevention (2011) CDC Grand Rounds: Chlamydia prevention: challenges and strategies for reducing disease burden and sequelae. MMWR - Morbidity & Mortality Weekly Report 60(12): 370-373.
