# Supplementary material for: Genital Chlamydia Prevalence in Europe and Non-European High Income Countries: Systematic Review and Meta-Analysis
Source: PLoS One. 2015 Jan 23;10(1):e0115753. doi: 10.1371/journal.pone.0115753 (PMC4304822; doi:10.1371/journal.pone.0115753)
Supplement: S2 Table — (PDF) [file pone.0115753.s003.pdf]

Table S2

Risk of bias in all studies

	Target population representative of country	Target population clearly defined	Source population representative of target	Sample size calculation	Adequate sample size achieved	Probability sampling	Respondents match target population	Response rate 70% limit	Response rate 60% limit	Standardised questionnaire	NAAT used	Appropriate analysis	Confidence intervals
EU/EEA countries													
Denmark 1998	−	?	?	?	?	+	−	−	−	+	+	+	+
Denmark 1999	−	+	−	−	?	+	−	−	−	+	+	+	+
Denmark 2001	?	+	+	?	?	+	−	−	−	+	+	+	+
Denmark 2002	?	?	?	?	?	+	?	−	−	+	+	+	+
Estonia 2008	?	+	+	+	?	+	−	−	−	+	+	+	+
France 2010	+	+	+	+	?	+	?	−	−	+	+	+	+
Germany 2012	+	+	+	+	?	+	+	−	+	+	+	+	+
Netherlands 2000	−	+	?	−	?	+	−	−	−	+	+	+	+
Netherlands 2005	+	+	?	+	+	+	−	−	−	+	+	+	+
Netherlands 2010	?	+	+	+	?	+	−	−	−	+	+	+	+
Norway 2005	−	+	?	?	?	+	−	−	−	+	+	+	+
Norway 2012	?	+	+	−	?	+	−	−	−	+	+	+	+
Slovenia 2004	+	+	+	−	?	+	−	−	+	+	+	+	+
Spain 2007	−	+	?	+	−	+	−	−	−	+	+	+	+
Sweden 1992	−	+	+	−	?	−	?	−	+	+	−	+	+
Sweden 1995	−	+	?	+	?	+	+	+	+	+	−	+	+
Sweden 2003	−	?	?	−	?	−	?	−	+	+	+	+	+
Sweden 2004	−	?	?	−	?	+	?	−	+	+	+	+	−

Sweden 2007	−	?	?	−	?	?	−	−	−	+	+	?	+
United Kingdom 2000a	−	+	?	−	?	+	−	−	−	+	+	+	+
United Kingdom 2000b	−	?	?	−	?	+	−	−	−	+	+	+	+
United Kingdom 2001	+	+	−	−	?	+	−	+	+	+	+	+	+
United Kingdom 2007	?	+	?	+	−	+	−	−	−	+	+	+	+
United Kingdom 2012	?	?	?	−	?	+	−	−	−	+	+	+	+

	Target population representative of country	Target population clearly defined	Source population representative of target	Sample size calculation	Adequate sample size achieved	Probability sampling	Respondents match target population	Response rate 70% limit	Response rate 60% limit	Standardised questionnaire	NAAT used	Appropriate analysis	Confidence intervals
Other European and high-income OECD countries													
Croatia 2011	+	+	?	+	?	+	−	−	−	+	+	+	+
Switzerland 2008	?	+	?	−	?	+	?	?	?	+	+	+	+
USA 2001	−	+	+	+	+	+	−	−	+	+	+	+	+
USA 2002a	?	+	?	−	?	+	+	−	−	+	+	+	−
USA 2002b	−	+	+	−	?	+	+	+	+	+	+	+	+
USA 2004	+	+	+	−	?	+	+	+	+	+	+	+	+
USA 2011	?	+	+	−	?	+	−	−	−	+	+	+	+
USA 2012	+	+	+	−	?	+	−	+	+	+	+	+	+
Canada 2002	−	?	?	−	?	−	?	−	−	+	+	+	−
Canada 2009	−	+	?	−	?	−	−	+	+	+	+	+	−
Australia 2003	−	?	?	−	?	−	+	−	−	+	+	+	+
Australia 2004	−	?	?	?	?	?	?	−	−	?	+	+	−
Australia 2006	?	+	+	+	−	+	−	−	−	+	+	+	+

Australia 2008	−	+	?	−	?	?	+	?	?	?	+	+	−
New Zealand 2002	−	+	?	+	−	+	−	+	+	+	+	+	+

Legend for Supplementary Figure 1

Response rate (defined as number tested/number asked to participate)
+ more than 80% + = 70-80% − less than 70% ? unclear/cannot be calculated (70% limit) OR
+ more than 80% + = 60-80% − less than 60% ? unclear/cannot be calculated (60% limit)
NAAT used and confidence intervals for positivity/prevalence estimates were included or could be calculated.
+ yes − no
All other items + adequate − inadequate ? unclear/not enough information provided
