# Supplementary material for: Genital Chlamydia Prevalence in Europe and Non-European High Income Countries: Systematic Review and Meta-Analysis
Source: PLoS One. 2015 Jan 23;10(1):e0115753. doi: 10.1371/journal.pone.0115753 (PMC4304822; doi:10.1371/journal.pone.0115753)
Supplement: S1 Text — (PDF) [file pone.0115753.s004.pdf]

Protocol

Population prevalence of *Chlamydia trachomatis* infection in the European Union, European Economic Area and other high income countries

A systematic review and meta-analysis

Protocol version 1.1 (September 2011)
Protocol version 2.0 (August 2012, protocol changes highlighted)
Protocol version 2.1 (technical editing 27.12.2013)

1. Background

Chlamydia trachomatis infection is the most commonly reported sexually transmitted infection in Europe [1]. Young adult women and men under the age of 25-30 years are the population group most likely to be infected [2]. In women, the bacteria can ascend from the endocervix, resulting in upper genital tract infection. This can cause pelvic inflammatory disease with potential sequelae of tubal factor infertility, ectopic pregnancy or chronic pelvic pain. *C. trachomatis* has been identified in the placenta of infected pregnant women [3] and infection during pregnancy is associated with prematurity [4]. Infection of the neonate during labour can lead to severe conjunctivitis and pneumonia [2]. In men, ascending chlamydia infection can lead to epididymitis. Additional complications that are more common in men than women include reactive arthritis and Reiter's syndrome. For both sexes, chlamydia infection increases susceptibility and transmission of HIV [5]. Complications of genital tract infection with *C. trachomatis* can impair quality of life in women [6] and result in substantial costs to the healthcare system [7].

Chlamydia infection is preventable and treatable [8].Chlamydia infection can be treated with antibiotics. A single dose of azithromycin or a seven day course of doxycycline is efficacious for short term microbiological cure of *C. trachomatis* in 97-98% of cases [9]. Partner notification and management are essential parts of chlamydia case management for identifying infected cases and preventing re-infection in the index case [10]. Most chlamydia infections are, however, asymptomatic or cause non-specific symptoms, which are often not recognised, particularly in women. Screening of people at high risk of infection is recommended in several European and other high income countries to identify and treat asymptomatic infections and to prevent long term complications [11-14].

Surveys of the population prevalence of *C. trachomatis* infections (chlamydia) can provide information for health policy decision makers about the need for measures to prevent and control infection. The least biased estimates of the prevalence of any condition at a particular time come from cross-sectional surveys of a representative sample of the general population (population-based surveys) [15]. Several large population-based surveys of chlamydia infection have been done in European Union (EU) Member States such as Great Britain [16] and the Netherlands [17] and other countries such as the USA [18]. Estimates of chlamydia prevalence vary between studies, even across countries with similar levels of social and economic development. Differences in estimates between countries could be real (representing differences in sexual behaviour patterns and chlamydia control efforts), but might also result from variations in study design and participation rates. A systematic review of chlamydia prevalence surveys would allow the available data to be collated and differences between studies to be investigated.

This systematic review is part of a project, Chlamydia Control in Europe, initiated, funded and conducted under a framework contract by the European Centre for Disease Prevention and Control (Framework Contract ECDC/2011/031). The main objective of the project was to provide information about Member States of the EU and European Economic Area (EEA). For this review, we will cover other countries in Europe and internationally to examine consistency and increase the generalisability of our findings.

2. Objective

To systematically review surveys estimating the prevalence of *C. trachomatis* infection in the general population of EU/EEA Member States and other high income countries.

3. Methods

3.1. Review questions

1. What is the prevalence of *C. trachomatis* infection in the general population of the EU/EEA Member States and other high income countries?
2. What is the distribution of chlamydia infection in different age, sex and ethnic groups?
3. What methodological features of cross-sectional studies influence estimates of *C. trachomatis* prevalence?

3.2. Inclusion criteria

Using the PICOS (population, intervention, comparison, outcome, study design) framework for defining systematic review questions:

3.2.a. Population

- General population of EU (http://europa.eu/about-eu/countries/member-countries/) and EEA (http://www.efta.int/eea) Member States, as of October 2011: Austria, Belgium, Bulgaria, Cyprus, Czech Republic, Denmark, Estonia, Finland, France, Germany, Greece, Hungary, Iceland, Ireland, Italy, Latvia, Liechtenstein, Lithuania, Luxembourg, Malta, Netherlands, Norway, Poland, Portugal, Romania, Slovakia, Slovenia, Spain, Sweden and United Kingdom;
- General population of non-EU/EEA European countries, the USA, Canada, Australia, and New Zealand. During the course of the review, before statistical analysis, we decided to include all high income countries, using the definition of the Organisation for Economic Co-operation and Development (OECD, http://www.oecd.org/tad/crc.htm);
- Adults and young people aged 13 years and over;
- Women and men.

3.2.b. Intervention

Not relevant to this review of observational studies

3.2.c. Comparison group

Not relevant to this review of observational studies

3.2.d. Outcome

C. trachomatis infection, defined as a positive result of diagnostic tests used by the study investigators.

3.2.e. Study design

Surveys using methods to obtain a representative sample of the general population or a whole country or sub-national region of a country in one of the following study designs:

- Cross-sectional surveys;
- Baseline survey in randomised controlled trials or cohort studies;
- Systematic review if original data were reported;

- Specimens taken from the urogenital-tract;

3.3. Exclusion criteria

- Countries other than those mentioned above;
- Serological studies and other studies sampling only from extra-genital sites;
- Narrative reviews about *C. trachomatis* that do not contain original data;
- Participant age below 13 years;
- Letters, commentaries and editorials.

3.4. Search strategy

3.4.a. Electronic databases

The following databases will be searched from January 1990 to October 2011. The search will be updated before starting statistical analysis. We will not apply any language restrictions:

- Ovid Medline;
- Embase;
- Popline;
- The Cochrane Library.

3.4.b. Search terms

We will use Medical Subject Headings (MeSH) and explosion search terms for searching Medline and corresponding thesaurus terms for other databases where available, combined using Boolean operators:

- *Chlamydia trachomatis* OR chlamydia infections (NOT *Chlamydophila pneumoniae* OR trachoma) AND
- Names of any eligible individual countries (including historical names from 1990 because 'German Federal Republic', 'German Democratic Republic', Czechoslovakia and Yugoslavia do not appear in the exploded search term) AND
- Prevalence

Search strategies for each database are shown in *Appendix 1*.

3.4.c. Additional searches

- Reference lists: if retrieved publications include source references for potential studies about the prevalence of *C. trachomatis* infections we will retrieve the originals;
- We will contact experts in the field to ask if they know of any additional publications not identified by the search strategy.

3.4.d. De-duplication

We will use Endnote bibliographic software for reference management. The following rules will be used to remove duplicate hits from the database:

1. Compare the title, or various combinations of author, year, secondary title, volume, issue and pages through the 'de-duplication' function;

2. Visually compare the full records of suspected duplicates;
3. Save duplicates in a separate database.

3.5. Selection of eligible studies

Two suitably qualified reviewers will screen titles and abstracts of articles identified by the search strategy independently, using a form to document potential eligibility. Any study selected as being potentially eligible by either reviewer, will be retained for review of the full text. Where no abstract is available electronically, and eligibility cannot be judged from the title alone, the full text of the article will be retrieved and screened. The abstracts of articles identified through additional searches will be reviewed in the same manner as those identified through database searches.

Data will be entered into Epidata (Epidata version 3.1, EpiData Association, Odense, Denmark). The items included in the screening form are listed in *Appendix 2*.

3.5.a. Retrieval of full-text articles

We will obtain the full text of articles or other documents reporting studies identified as being potentially eligible for inclusion. We will make every effort to locate documents through internet downloads, inter-library loans and contacting authors of reviews citing potentially eligible documents. We intend to have articles translated if necessary to confirm or refute eligibility.

3.5.b. Selection of studies for final inclusion

The two independent reviewers will examine full text articles using a more detailed form and compare their lists of studies eligible for inclusion. Studies identified by both reviewers as being eligible for inclusion and having adequate data for extraction will be included in the review. Where there are discrepancies, the reasons for these will be discussed and a decision about inclusion reached by consensus. If there is no agreement, a third independent reviewer will adjudicate to make a final decision about eligibility. The selection of studies is described in a flow chart, and will be included in the publication of the review results. A version of the proposed flowchart is included as *Appendix 3.*

3.5.c. Selecting a population for each country

We aim to identify surveys in each eligible country, carried out in a sample that is representative of the general population. We consider the following groups as part of the general population: school students if the sampling frame included all schools in the country or in a sub-national geographic region of a country; and military recruits in countries with compulsory military conscription.

As part of the overall project, for EU/EEA Member States only, we aim to catalogue surveys estimating levels of chlamydia infection in other settings, such as health-care facilities or outreach studies. We defined categories, according to setting and population (*Appendix 4*).

3.6. Data extraction

Two appropriately qualified reviewers will extract and enter data independently from each included study into Epidata (*Appendix 5*).

Articles in languages other than English will be either translated first and then duplicate data extraction conducted as above or, if there are two reviewers who understand the language of publication, they will extract the data directly.

The two files will be compared using the validation function available in Epidata. Discrepancies in data extraction or data entry will be resolved by consensus. If there is no agreement a third independent reviewer will adjudicate to make a final decision.

Some studies may be excluded at the data entry stage if it became apparent that inclusion criteria are not met or there is not enough information in the documents to extract the required data.

3.6.a. Data extraction forms

The following outcomes will be extracted using Epidata (detailed list of items in *Appendix 2b*):

- Study design;
- Country;
- Population setting: general population of whole country or sub-national population of area;
- Study population: sexually experienced only or all participants;
- Sex, age and ethnic group of participating individuals;
- Social-demographic characteristics, specified if not concerning the general population;
- Numbers eligible, invited, accepting participation, providing samples, samples tested, number of samples included in analysis;
- Numbers excluded, with reasons;
- Diagnostic test method;
- Numbers with positive *C. trachomatis* test result;
- Authors' estimated prevalence and 95% confidence intervals (CI);
- Comparison of responders vs. non-responders, if reported;
- Methodological and reporting quality (Adapted from Boyle, Guidelines for evaluation of prevalence studies) [15] (*Appendix 6*).

3.7. Data analysis

3.7.a. Descriptive analysis

Review questions 1 and 2: C. trachomatis prevalence estimates

We will tabulate estimates of prevalence from each study. Where complex sampling methods had been used we will use the 95% CI presented in published papers. Where simple random sampling has been done and data are available, we will calculate *C. trachomatis* prevalence (with binomial 95% CI) for the available sex, age and ethnic groups.

We will display estimates in forest plots to show the point estimate and confidence intervals for each study.

We will calculate response rates to each survey, based on data provided about the eligible population. Using algorithms defined by the Council of American Survey Research Organizations (CASRO) [19, 20] we will exclude respondents who were ill, away from home or unable to speak English, where possible. We will use authors' reported response rates in complex surveys involving post-stratification weighting.

3.7.b. Statistical analysis

We will use meta-analysis to combine estimates of chlamydia prevalence where appropriate and to examine evidence of between study heterogeneity.

We will use the I^2 statistic to describe the percentage of the variability of results between studies that is due to factors other than random variation [21]. As a guide, I^2 values above 25%, 50% and 75% are suggested as evidence of mild, moderate and severe between study heterogeneity. Where there is evidence of moderate or severe heterogeneity, we will explore reasons for this by stratification or, if enough studies are available, by meta-regression.

Review question 3

We will examine the influence of setting, response rate, and other study characteristics using meta-regression to examine reasons for heterogeneity. We will examine the linear association between estimated chlamydia prevalence and the calculated response rate.

All analyses will be done using Stata statistical software (Stata 11, StataCorp, Austin, Texas, USA).

4. Report writing

Reports will be written following preferred reporting items for reporting of systematic reviews and meta-analyses (PRISMA) guidelines [22].

5. References

1. European Centre for Disease Prevention and Control. Sexually transmitted infections in Europe 2011. Stockholm: European Centre for Disease Prevention and Control; 2013.

2. Holmes KK, Sparling PF, Stamm WE, Piot P, Wasserheit JN, Corey L et al. Sexually Transmitted Diseases. New York: McGraw-Hill; 2008.

3. Rours GI, de Krijger RR, Ott A, Willemse HF, de GR, Zimmermann LJ et al. Chlamydia trachomatis and placental inflammation in early preterm delivery. Eur J Epidemiol. 2011;26(5):421-8. doi:10.1007/s10654-011-9569-2.

4. Rours GI, Duijts L, Moll HA, Arends LR, de Groot R, Jaddoe VW et al. Chlamydia trachomatis infection during pregnancy associated with preterm delivery: a population-based prospective cohort study. Eur J Epidemiol. 2011;26(6):493-502. doi:10.1007/s10654-011-9586-1.

5. Cohen MS, Hoffman IF, Royce RA, Kazembe P, Dyer JR, Daly CC et al. Reduction of concentration of HIV-1 in semen after treatment of urethritis: implications for prevention of sexual transmission of HIV-1. Lancet. 1997;349(9069):1868-73.

6. Smith KJ, Tsevat J, Ness RB, Wiesenfeld HC, Roberts MS. Quality of life utilities for pelvic inflammatory disease health states. Sex Transm Dis. 2008;35(3):307-11. doi:10.1097/OLQ.0b013e31815b07dd.

7. Owusu-Edusei K, Jr., Chesson HW, Gift TL, Tao G, Mahajan R, Ocfemia MC et al. The estimated direct medical cost of selected sexually transmitted infections in the United States, 2008. Sex Transm Dis. 2013;40(3):197-201. doi:10.1097/OLQ.0b013e318285c6d2.

8. Ward H, Fredlund H, Gotz H, Goulet V, Robinson A, Uuskula A. ECDC Guidance. Chlamydia control in Europe, June 2009. Stockholm: European Centre for Disease Prevention and Control; 2009.

9. Lau C-Y, Qureshi AK. Azithromycin versus doxycycline for genital chlamydial infections: a meta-analysis of randomized clinical trials. Sex Transm Dis 2002;29(9):497-502.

10. Ferreira A, Young T, Mathews C, Zunza M, Low N. Strategies for parttner notification for sexually transmitted infections, including HIV. Cochrane Database Syst Rev 2013; Issue 10. Art. No.: CD002843. DOI: 10.1002/14651858.CD002843.pub2.

11. Centers for Disease Control and Prevention. Sexually Transmitted Diseases Treatment Guidelines, 2010. Morbid Mortal Weekly Rep. 2010;59(RR-12):44-5.

12. Royal Australian College of General Practitioners. Guidelines for Preventive Activities in General Practice. Melbourne: RACGP; 2007.

13. National Chlamydia Screening Programme. National Chlamydia Screening Programme, England. Core requirements. 5th Edition, update 2 - August 2010. London: Health Protection Agency; 2010.

14. Meyers DS, Halvorson H, Luckhaupt S, Force USPST. Screening for chlamydial infection: an evidence update for the U.S. Preventive Services Task Force. Ann Intern Med. 2007;147(2):135-42.

15. Boyle MH. Guidelines for evaluating prevalence studies. Evidence Based Mental Health. 1998;1(2):37-9.

16. Fenton KA, Korovessis C, Johnson AM, McCadden A, McManus S, Wellings K et al. Sexual behaviour in Britain: reported sexually transmitted infections and prevalent genital *Chlamydia trachomatis* infection. Lancet 2001;358(9296):1851-4.

17. van Bergen J, Götz H, Richardus J, Hoebe C, Broer J, Coenen A. Prevalence of urogenital Chlamydia trachomatis increases significantly with level of urbanisation and suggests targeted screening approaches: results from the first national population based study in the Netherlands. Sex Transm Infect. 2005;81(1):17-23.

18. Miller WC, Ford CA, Morris M, Handcock MS, Schmitz JL, Hobbs MM et al. Prevalence of chlamydial and gonococcal infections among young adults in the United States. JAMA 2004;291(18):2229-36.

19. Erens B, McManus S, Field J, Korovessis C, Johnson AM, Fenton K et al. National Survey of Sexual Attitudes and Lifestyles II: Technical Report. London: National Centre for Social Research; 2001.

20. Council of American Survey Research Organizations. On the definition of response rates. www.casro.org. 1982. www.casro.org/resource/resmgr/docs/casro_on_definitions_of_resp.pdf.

21. Higgins J, Thompson SG. Quantifying heterogeneity in a meta-analysis. Stat Med 2002;21(11):1539-58.

22. Moher D, Liberati A, Tetzlaff J, Altman DG, The Prisma Group. Preferred Reporting Items for Systematic Reviews and Meta-Analyses: The PRISMA Statement. PLoS Med. 2009;6(7):e1000097. doi:10.1371/journal.pmed.1000097.

Appendix 1: Search strategies

Ovid Medline

1.	('chlamydia infections' not ('chlamydophila pneumoniae' or trachoma or 'lymphogranuloma venereum')).mp.
2.	prevalence.mp.
3.	europe/ or exp austria/ or exp belgium/ or europe, eastern/ or exp baltic states/ or exp bulgaria/ or exp czech republic/ or exp hungary/ or exp poland/ or exp romania/ or exp slovakia/ or exp slovenia/ or exp yugoslavia/ or exp finland/ or exp france/ or exp germany/ or exp great britain/ or exp greece/ or exp iceland/ or exp ireland/ or exp italy/ or exp liechtenstein/ or exp luxembourg/ or exp mediterranean region/ or exp netherlands/ or exp portugal/ or exp scandinavia/ or exp spain/ or exp switzerland/ or czechoslovakia/ or european union/ canada/ or united states/ or australia/ or new zealand/ japan/ or korea/ or israel/
4.	*prevalence.mp. or mass screening/mt*
5.	*(austria or belgium or estonia or latvia or lithuania or bulgaria or czech republic or hungary or poland or romania or slovakia or slovenia or yugoslavia or finland or france or germany or great Britain or greece or iceland or ireland or italy or liechtenstein or luxembourg or malta or cyprus or netherlands or portugal or norway or sweden or denmark or spain or switzerland or czechoslovakia).mp.*
6.	1 and 2 and 3
7.	1 and 4 and 5
8.	6 and 7
9.	Limit 8 to (humans and yr="1990 –Current")

Limits: 1990-current, humans; homepage: http://ovidsp.tx.ovid.com/

Embase

1.	'chlamydiasis'/exp NOT ('lymphogranuloma venereum'/exp OR 'trachoma'/exp) AND [humans]/lim AND [embase]/lim AND [1990-2012]/py
2.	'prevalence'/exp NOT ('human immunodeficiency virus prevalence'/exp OR 'seroprevalence'/exp OR 'parasite prevalence'/exp) AND [humans]/lim AND [embase]/lim AND [1990-2012]/py
3.	'europe'/de OR 'eastern europe'/de OR 'western europe'/de OR 'austria'/exp OR 'baltic states'/exp OR 'belgium'/exp OR 'bulgaria'/exp OR 'cyprus'/exp OR 'czech republic'/exp OR 'czechoslovakia'/exp OR 'france'/exp OR 'germany'/exp OR 'greece'/exp OR 'hungary'/exp OR 'ireland'/exp OR 'italy'/exp OR 'luxembourg'/exp OR 'malta'/exp OR 'netherlands'/exp OR 'poland'/exp OR 'portugal'/exp OR 'romania'/exp OR 'slovakia'/exp OR 'slovenia'/exp OR 'yugoslavia'/exp OR 'scandinavia'/exp OR 'spain'/exp OR 'united kingdom'/exp OR 'switzerland'/exp OR 'iceland'/exp OR 'liechtenstein'/exp OR 'australia'/de OR 'canada'/de OR 'new zealand'/de OR 'united states'/de OR 'japan'/de OR 'korea'/de OR 'israel'/de AND [humans]/lim AND [embase]/lim AND [1990-2013]/py
4.	#1 AND #2 AND #3

Limits: 1990-current, humans, search in Embase only;; Homepage: http://www.embase.com

Popline

	CHLAMYDIA & PREVALENCE & (EUROPE / 'EUROPEAN UNION' / AUSTRIA / BELGIUM / BULGARIA / CYPRUS / 'CZECH REPUBLIC' / CZECHOSLOVAKIA / DENMARK / ESTONIA / FINLAND / FRANCE / GERMANY / 'GERMAN DEMOCRATIC REPUBLIC' / 'FEDERAL REPUBLIC OF GERMANY' / GREECE / HUNGARY / IRELAND / ITALY / LATVIA / LITHUANIA / LUXEMBOURG / MALTA / NETHERLANDS / POLAND / PORTUGAL / ROMANIA / SLOVAKIA / SLOVENIA / SPAIN / SWEDEN / 'UNITED KINGDOM' / YUGOSLAVIA / SWITZERLAND / NORWAY / ICELAND / LIECHTENSTEIN / AUSTRALIA / CANADA / 'NEW ZEALAND' / 'UNITED STATES OF AMERICA'/ JAPAN / KOREA / ISRAEL) NOT TRACHOMA

Limits: search limited to title/keywords; Endnote Import Filter: http://db.jhuccp.org/popinform/basic.html; homepage: http://www.popline.org

The Cochrane Library

1.	MeSH descriptor Chlamydia Infections explode all trees
2.	MeSH descriptor Prevalence explode all trees
3.	MeSH descriptor Europe explode all trees
4.	MeSH descriptor (AUSTRALIA OR CANADA OR NEW ZEALAND OR UNITED STATES OR JAPAN OR KOREA OR ISRAEL)
6.	#1 AND #2 AND #3 OR #4, limit to 1990 – 2012

Homepage: http://onlinelibrary.wiley.com/o/cochrane/cochrane_search_fs.html

Appendix 2: Screening form for study eligibility

Endnote ID: ___

First author: ___

Checklist completed by: __

Inclusion for data extraction?

- Yes;
- Provisional;
- No;
- No, but interesting (retain for discussion or future use)

Study design:

- Cross-sectional study
- Randomised controlled trial
- Cohort study
- Other: ___

Outcomes described for following population (Used for categories in Appendix 5)

- General population
- General population of a sub-national geographical area
- Population other than in health care settings e.g. schools, institutions, prisons: ___
- Population in health care settings
- Population in genitourinary medicine/sexually transmitted diseases clinic settings
- Population of a specific subgroup e.g. commercial sex worker, men who have sex with men
- People with comorbidity
- Other, describe: ___

Reason for exclusion

- Other than general population if general population are available for this country
- Topic not relevant
- Country not of interest
- Narrative review
- Specimen not from uro-genital tract
- Age under 13 years
- If USA/CAN/AUS/NZ (or other OECD high income countries) not general population
- Study type not relevant
- Other: ___

Appendix 3: Flow chart of the selection process

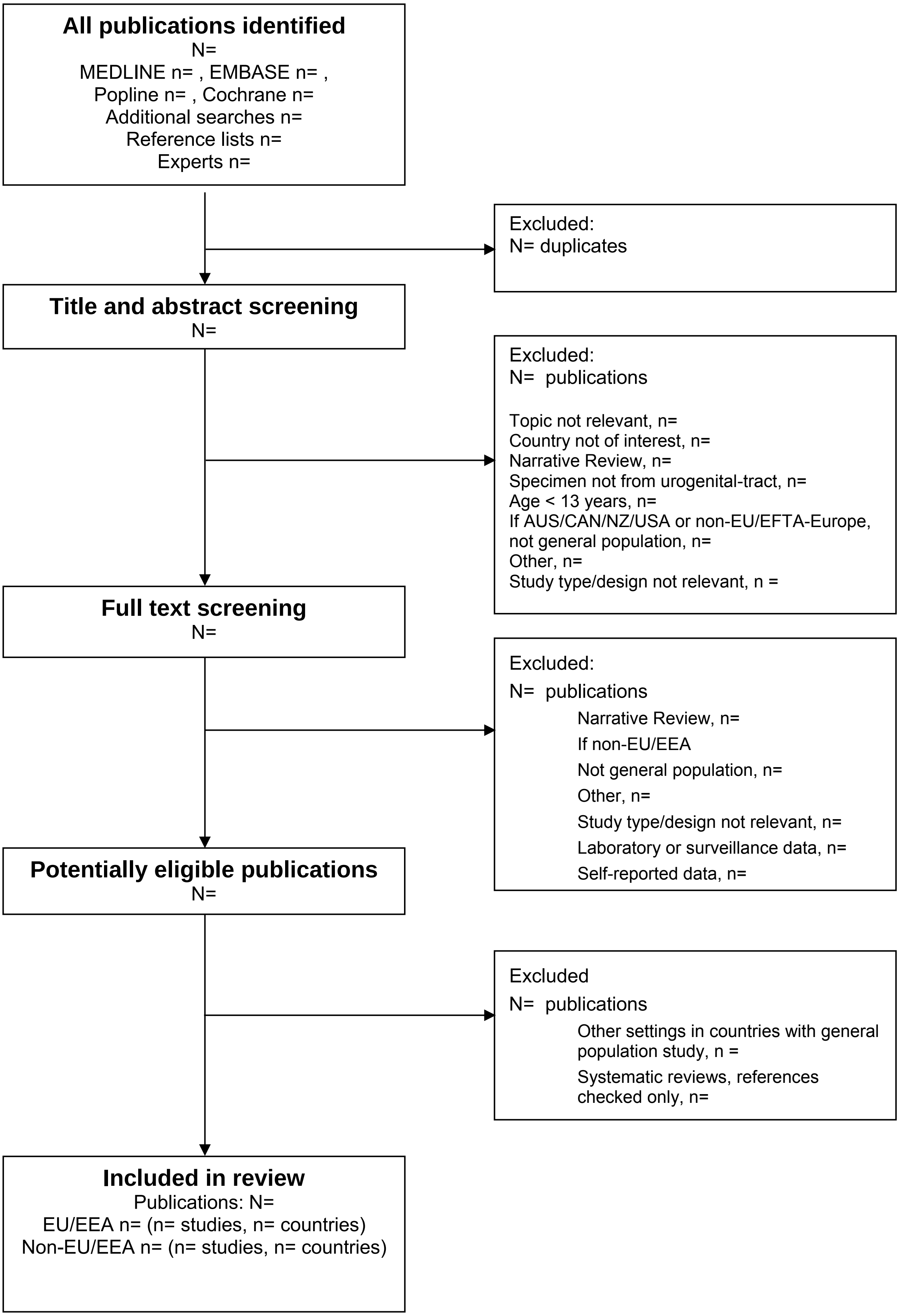

Appendix 4: Categories of study populations in studies with non-population based sampling methods

- Category 1A: defined random sample of the general population nationally, fulfilling all relevant criteria to minimise risk of bias (Appendix 5)
- Category 1B: defined random sample of the general population nationally, NOT fulfilling all relevant criteria to minimise risk of bias
- Category 2A: defined random sample of the general population of a sub-national geographical region, fulfilling all relevant criteria to minimise risk of bias
- Category 2B: defined random sample of the general population of a a sub-national geographical region, NOT fulfilling all relevant criteria to minimise risk of bias
- Category 3A: random or consecutive sample of a population similar to the general population and not in health care settings, e.g. Schools, Universities, sport-clubs fulfilling all relevant criteria to minimise risk of bias
- Category 3B: random or consecutive sample of a population similar to the general population and not in health care settings, e.g. Schools, Universities, sport-clubs NOT fulfilling all relevant criteria to minimise risk of bias
- Category 4A: random or consecutive sample of attenders at a non-GUM clinic healthcare setting, fulfilling all the relevant criteria to minimise risk of bias
- Category 4B: random or consecutive sample of attenders at a non-GUM clinic healthcare setting, NOT fulfilling all the relevant criteria to minimise risk of bias
- Category 5A: random or consecutive sample from a GUM clinic, fulfilling all the relevant criteria to minimise risk of bias
- Category 5B: data from a GUM-clinic, NOT fulfilling all the relevant criteria to minimise risk of bias
- Category 6A: sample of a specific subgroup at risk e.g. CSW, HIV-positive persons, MSM, fulfilling all the relevant criteria to minimise risk of bias
- Category 6B: sample of a specific subgroup at risk e.g. CSW, HIV-positive persons, MSM, NOT fulfilling all the relevant criteria to minimise risk of bias
- Category 7A: sample of patients with co-morbidity, such as other STD, symptoms of urethritis or PID, infertility, EUG, abortion fulfilling all the relevant criteria to minimise risk of bias
- Category 7B: sample of patients with co-morbidity, such as other STD, symptoms of urethritis or PID, infertility, EUG, abortion NOT fulfilling all the relevant criteria to minimise risk of bias
- Category 8A: other population, such as laboratory reports, specific subgroups which don’t fit into the categories above, fulfilling all the relevant criteria to minimise risk of bias
- Category 8B: other population, such as laboratory reports, specific subgroups which don’t fit into the categories above, NOT fulfilling all the relevant criteria to minimise risk of bias

Appendix 5: Data extraction items

Study design:

- Cross-sectional study
- Randomised controlled trial
- Cohort study
- Other, describe: ___

Inclusion criteria, describe: ___

Exclusion criteria, describe: ___

Select country from the list below:

Austria; Belgium; Bulgaria; Cyprus; Czech Republic; Denmark; Estonia; Finland; France; Great Britain; Germany; Greece; Hungary; Ireland; Italy; Latvia; Lithuania; Luxembourg; Malta; Netherlands; Poland; Portugal; Romania; Slovakia; Slovenia; Spain; Sweden; Iceland; Liechtenstein; Norway; Switzerland; Australia; New Zealand; USA; Canada; other country, describe: ___

Methods

Setting: General population of whole country or general population of a sub-national geographical area

Describe methods and recruitment procedure: ___

Initial approach:

- Mail
- Telephone
- Internet
- Personal contact in household
- Personal contact in health care settings
- Other, describe:

Date of study from ___ until___

Method of *C. trachomatis* detection:

- Nucleic acid amplification test (NAAT) urine;
- NAAT swab
- Enzyme linked immunosorbent assay (ELISA) urine
- ELISA swab
- Immunofluorescence
- Culture
- Other, describe: ___
- Unclear

Is ethical committee approval reported? Yes; No; Not applicable; Unclear

Informed consent; Yes; No; Unclear

Ethnic group:

- White
- Black including Caribbean, African
- Indian, Pakistani and Bangladeshi
- Chinese

- Other Asian
- North African, Arab, Iranian
- Romani
- Not reported/unclear
- Other including unknown, describe: ___

Sampling method:

- Simple random sampling
- Stratified random sampling
- Multistage stratified random sampling
- Consecutive sample
- Convenience sample
- Unclear
- Other
- Describe sampling method: ___

Outcomes reported in the study:

- Prevalence
- Response rate

Outcomes reported for following subgroups (each outcome numbered):

- Age groups combined
- Age groups stratified
- Men and women combined
- Men and women separate
- Men only
- Women only
- Ethnic groups separate
- Certain ethnic group only
- Number of lifetime partners any categorisation
- Not specified
- Others, not covered above: ___

Description of target population: ___

Description of source population: ___

Reported for: Women and men combined; women only; men only

Total number of eligible people__

Total number of people able to participate__

Total number of people asked to participate /sent questionnaire to__

Total number of people agreed to participate/ filled in questionnaire__

Total number of people asked for sample__

Total number of people providing sample__

Total number of samples tested__

Total number of test results included in analysis__

Do these numbers reported above add up logically? Yes; No; Unclear

How were missing data handled? Describe: ___

Age range of participants in overall sample lower limit __; upper limit__

Has any multivariable analysis been done to examine factors associated with Chlamydia? Yes, No; Unclear; If yes describe characteristics analysed: ___

Numerical outcomes

Endnote ID:___

Outcome number

Describe group: ___

Country number for this outcome

Setting

- General population
- General population of a sub-national geographical area
- Describe setting: ___

Sex: Women and men combined; Only women; Only men

Age group: (lower limit to upper limit of age group, write ’99’ if unknown)

Specimen handed in:

- Urine
- Swab
- Unclear/not reported
- Other, describe:___

Collection method

- Specimen collected by physician
- Self-collected specimen

Ethnic group

- White
- Black, including Caribbean, African
- Indian, Pakistani and Bangladeshi
- Chinese
- Other Asian
- North African, Arab, Iranian
- Romani
- Other, describe: ___
- Not reported/unclear

Raw data, if reported (write 999999 if not reported)

- Number tested positive
- Weighted number tested
- Un-weighted number tested

Outcome (for each reported numbered outcome)

- Prevalence and 95%CI (lower limit, upper limit)
- Response rate and 95%CI (lower limit, upper limit)

Appendix 6: Risk of bias assessment

We will use a list of published items by Boyle for assessing the methodological risk of bias in prevalence surveys [15]. We will use the published criteria to determine whether the risk of bias has been addressed adequately, inadequately, or if there was insufficient information to judge. For some items, we developed our own criteria, reviewers assess methods independently and agree on category by consensus or adjudication.

Items assessed

Item and explanation	Assessment	Criteria
*Is the target population clearly defined?<br>Target population is the population to which the main results of the study will be extrapolated.	Adequate	Target population is defined by shared characteristics, such as age, sex, residency, sexual activity
	Inadequate	Characteristics of target population not described
	Unclear	Characteristics insufficiently defined
Is the source population clearly defined?†<br>Source population is the population from which investigators selected the random sample.	Adequate	Characteristics of the source population are clearly defined, e.g. by age, sex, residency, sexual activity
	Inadequate	Characteristics of source population not described
	Unclear	Characteristics insufficiently defined
*Was probability sampling used to select the sample?	Adequate	Simple, stratified or multistage random sampling methods described
	Inadequate	Convenience sample or other non-random sampling method described
	Unclear	Methods not described in sufficient detail to determine if probability sampling used
Is the source population an adequate sample of the target population? †	Yes	If you can compare data about each and decide that there are no substantial differences, or the authors describe them as being similar
	No	If you or the authors conclude that there are important differences between the source and the target population
	Unclear	No description or unclear if there are important differences
Are the socio-demographic	Adequate	Comparison done and socio-demographic

characteristics of responders and non-responders similar?*		characteristics are described as ‘similar’ or no important differences observed
	Inadequate	No comparison done, or comparison shows important differences between responders and non-responders
	Unclear	Insufficient information to decide
Sample size calculation described?[†]	Yes	A sample size calculation is described to show acceptable precision
	No	No sample size calculation
	Unclear	Insufficient information provided
Adequate sample size achieved?[†]	Yes	Achieved sample size is similar to the sample size calculation
	Unclear, probably yes	No sample size calculation described, but precision of primary outcome judged acceptable
	Unclear, probably no	No sample size calculation described, but precision of primary outcome judged unacceptable
Response rate*	≥80%	Described by Boyle as acceptable [15]
Number tested/ Number asked to participate or sent questionnaire to. If other numbers are used to calculate the response rate, do not calculate the response rate.	70-79%	Categories defined by reviewers to describe response rates
	<70%	
Valid standardised questionnaire for data collection used?*	Adequate	Authors state that questionnaires for data like age, sex and risk behaviour are valid for all participants
	Inadequate	Authors state that different questionnaires used for different study groups, e.g. by age, sex and risk behaviour, or non-validated
	Unclear	Insufficient information provided
NAAT used for *C. trachomatis* detection?[†]	Yes	Includes: PCR (including RT-PCR, transcription mediated amplification), branched DNA tests, ligase chain reaction, strand displacement analysis

	No	Other diagnostic test described
	Not described	No information given about test used
Special features of sampling design were accounted by the use of special statistical methods?* Special statistical methods include weighting procedures to adjust for sampling probabilities.	Adequate	Either, special features were accounted for by appropriate statistical methods, or special methods were not needed because simple random sampling used.
	Inadequate	Complex sampling used but not accounted for by appropriate statistical methods
	Unclear	
Confidence intervals included?*	Yes	Reported by authors
	No	Not reported by authors
Data provided to calculate confidence interval?*	Yes	Simple random sampling and raw numbers (positive tests/total number of test results) provided
	No	Simple random sampling but no raw numbers
	Not relevant	Complex sampling method with confidence intervals calculated by authors

***** Items included in assessment tool and criteria for assessment adapted from descriptions by Boyle [15];

† Items added for this systematic review and criteria developed by review team.
